# A gigantic bizarre marine turtle (Testudines: Chelonioidea) from the Middle Campanian (Late Cretaceous) of South-western Europe

**DOI:** 10.1038/s41598-022-22619-w

**Published:** 2022-11-17

**Authors:** Oscar Castillo-Visa, Àngel H. Luján, Àngel Galobart, Albert Sellés

**Affiliations:** 1 Institut Català de Paleontologia Miquel Crusafont, Universitat Autònoma de Barcelona, Edifici ICTA-ICP, c/ Columnes s/n, Campus de la UAB, 08193 Cerdanyola del Vallès, Barcelona Spain; 2 Museu de la Conca Dellà, c/ Museu 4, 25650 Isona, Lleida Spain; 3 Department of Geological Sciences, Faculty of Sciences, Masaryk University, Kotlářská 267/2, 611 37 Brno, Czech Republic

**Keywords:** Palaeontology, Phylogenetics, Taxonomy, Evolution

## Abstract

Marine turtles were common in the subtropical Upper Cretaceous epi-continental seas that once washed the coasts of the ancient European archipelago. But unlike its contemporaneous faunas from North America, in Europe no taxon surpassed the 1.5 m shell-length. Here, the remains of a new large marine turtle, *Leviathanochelys aenigmatica* gen. et sp. nov., from the Middle Campanian of the Southern Pyrenees are described. Anatomical and histological evidence concur in identifying the specimen as a basal chelonioid. The new taxon autapomorphically differs from other marine turtles by possessing an additional process on the anteromedial side of the pelvis, and an acetabulum directed strongly ventrally. Based on the pelvis size, it is likely that *Leviathanochelys* was as large as *Archelon*, thus becoming one of the largest marine turtles found to ever exist. The large body size of the new taxon could have evolved as a response to the unique habitat conditions of the European Cretaceous archipelago seas. The presence of the accessory pubic process further suggests the occurrence of an additional insertion point of the *Musculus rectus abdominis*, which together with the paleohistologic evidences support the hypothesis that the new taxon had an open marine pelagic lifestyle.

## Introduction

Pan-Chelonioidea is a monophyletic clade of cryptodiran Testudines (if considering Protostegidae at the base of the superfamily) that comprises both extinct and extant marine turtles, including the largest turtles that have ever sailed the seas such as the protostegids *Archelon* or *Protostega*. The clade is characterized by having several anatomical adaptations to marine lifestyle, such as the modification of the autopodials in paddle-like limbs^1,2^, cranial modification for exceeding-salt removal^3^, and the reduction of the shell ossification, together with the development of fontanelles in both carapace and plastron^4^.

According to the most recent analyses^5^, the superfamily Chelonioidea only includes Dermochelyidae and Cheloniidae, while the extinct Toxochelyidae and Ctenochelyidae represent stem chelonioids. Anyhow, all of these groups emerged during the Early Cretaceous and peaked in diversity during the Latest Cretaceous^4,6–8^. On the other hand, the phylogenetically controversial protostegids^6^ experienced a rapid radiation during the Early Cretaceous, becoming globally widespread, ecologically specialized and taxonomically diverse during said period^9,10^, but become extinct at the end of the Mesozoic era. It is worth noting that gigantic forms mainly occurred during the Campanian in the Western Interior Sea of North America^11^, to the exception of the Moroccan *Ocepechelon*^12^, the Jordanian *Gigantatypus*^13^, and material from Eastern Europe referred to *Protostega gigas*^14^. The achievement of such large body size might be likely reflecting some unique habitat conditions or the selection towards large sizes by predatory pressure^15,16^.

**Figure 1:**
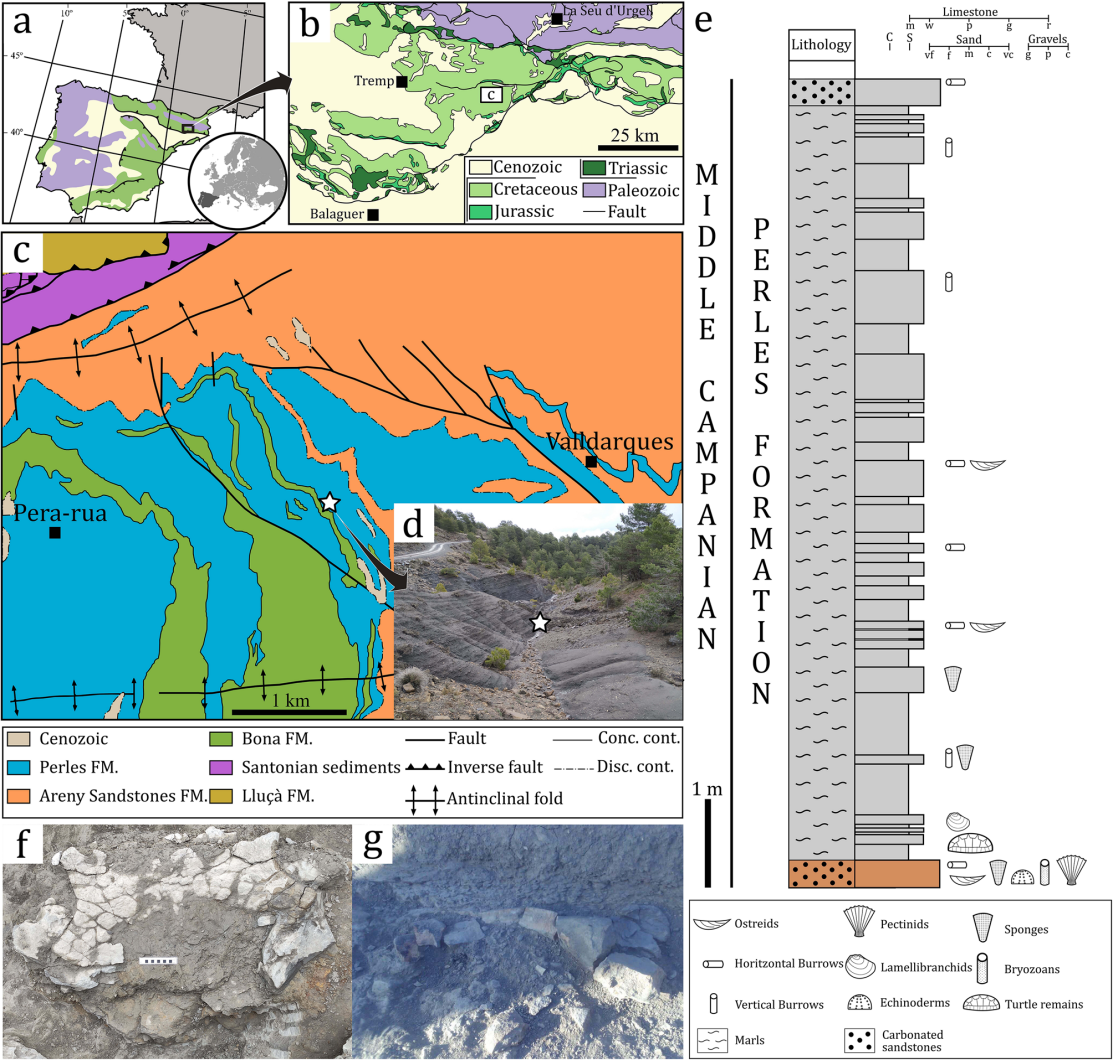
Geographic and geological situation of Cal Torrades. The Cal Torrades fossil locality location, respect: (**a**) the Iberian Peninsula; and (**b**) the Eastern Pyrenees. (**c**) Simplified geological map including the locality (white star). (**d**) Field capture of Cal Torrades outcrop, marking with the star the location of the fossil remains: (**e**) pelvis; and (**f**) ilium. (**g**) Locality stratigraphic column with the geological materials and fossil remains. Modified from Costantino and Angelini^26^, Vidal^27^ and free access digital maps of the Institut de Cartografia i Geologia de Catalunya (ICGC; http://www.icc.cat/vissir3/).

Although European marine deposits have yielded a relatively good fossil record of Late Cretaceous chelonioids^17,18^, the reports of large taxa are extremely rare^19–23^. With up to 1.5 m of shell-length, *Allopleuron hoffmanni* is, to date, the largest Late Cretaceous chelonioid in Europe^24,25^. Here, we report a new gigantic basal chelonioid, represented by a posterior region of the carapace and a partial pelvic girdle from the Middle Campanian marine deposits of Southern Pyrenees (Fig. 1). Despite the fragmentary nature of the specimen, its inferred body size rivals with that of *Archelon*, confirming the occurrence of colossal marine turtles in the Late Cretaceous European seas.

## Geological setting

During the last 20 years, our knowledge on the Campanian–Maastrichtian vertebrates from the Southern Pyrenees has increased significantly^28,29^. However, this is mainly restricted to terrestrial environments, and therefore little is known about the marine tetrapods that once inhabited the marine realm of this part of the Iberian Peninsula. Subsequently, here we describe the first locality yielding marine vertebrate remains from the end-Cretaceous of the Southern Pyrenees.

Discovered on July 2016, the locality of Cal Torrades is placed between the Serra d’Aubenç and the Serra de Carreu, outcropping near the small villages of Pera-Rua and Valldarques (Alt Urgell County, Southern Pyrenees; Fig. 1a–e). From a geological viewpoint, Cal Torrades is situated within the lower part of the Perles Formation, whose main lithologic components consist of marls and marly limestones^30,31^. The lower part of the Perles Formation contains abundant invertebrate macro (—e.g. echinoderms, sponges, lamellibranch bivalves) and microfossils (hyaline benthonic and planktonic foraminifera^32^, the analyses of which –mainly the benthonic foraminifera assemblage (see^33^)—allow to establish the age of the new fossil locality as Middle Campanian.

The stratigraphic section at Cal Torrades consists of an alternation between grey marls and sandstone levels (Fig. 1c–e). The locality rests upon a carbonated sandstone bar of 5 m in thickness, whose uppermost part is the base of the studied section (Fig. 1d,e). It contains abundant invertebrate fossil remains such as well-preserved ostreids, pectinid bivalves, hexactinellid sponges, echinoderms, horizontal burrows and ramified bryozoans. The chelonioid remains were found 20 cm over the top of the sandstone base (Fig. 1c–g). A nearly uniform sequence of marls is developed above the sandstone layer. Overall, the stratigraphic section can be defined as slight coarsening-upward sequence (Fig. 1e). The occurrence of a well-developed marly sequence, locally alternated with fine sandstone levels, likely indicates a low energetic offshore depositional environment. On the other hand, the presence of a thick sandstone bar with abundant organisms at the base of the stratigraphic section, rather suggests a relatively low water column. The combination of geological and fossiliferous evidence allows inferring a transgressive depositional sequence, from a near shore environment to a complete offshore environment.

Although a more detailed study is required, preliminary interpretations suggest that the sedimentological sequence at Cal Torrades may represent one of the last transgressive pulses, before the regressive sequence represented by the Upper Cretaceous Gresos d’Areny Formation^29^ in the Southern Pyrenees. The benthonic macroforaminifera assemblage recovered in the strata immediately above to the Perles Formation suggests a bathymetric depth between 20 and 80 m^34–36^. This interpretation concurs with the absence of high-energy sedimentary structures at the outcrop, suggesting that the depositional environment would be, at least, under the storm-affectation level. Thereby, it is most likely that the sedimentological sequence at the Cal Torrades locality was established in a marine mid-ramp environment, as well as its lateral equivalent formations (e.g. Terradets Formation; see^32,35^).

## Results

### Systematic palaeontology

Testudines Batsch, 1788.

Cryptodira Cope, 1868.

Chelonioidea Baur, 1893.

*Leviathanochelys aenigmatica* gen. et sp. nov.

urn:lsid:zoobank.org:act:28CDDCDB-AC31-45B6-98B1-448E3282A041.

urn:lsid:zoobank.org:act:2B8F389C-6437-450F-812C-E7CD8EFF07A2.

### Etymology

The generic name is composed of the following words: *Leviathan*, in reference to the Biblical marine beast, in allusion to the body size of the new species; and *chelys,* Latinized name from the ancient Greek χέλυς (“khélūs” meaning turtle in feminine gender). The specific nomination *aenigmatica*, Latinized adjective from the Greek noun αἴνιγμα (“aínigma” meaning enigma, conundrum or riddle) is in reference to the peculiar anatomical characteristics of its pelvis and carapace.

### Holotype

MCD9884. Posterior portion of the carapace including the neural plates 5–8, both left and right fragmentary costals 5–8 and a putative vertebral centra, nearly unidentifiable (MCD9884a); and a partial pelvic girdle, including: the left pubis (MCD9884b); right pubis (MCD9884c); left ischium (MCD9884d); right ischium (MCD9884e); left ilium (MCD9884f); and right ilium (MCD9884g).

### Type locality and age

Cal Torrades, Coll de Nargó (Lleida Province, Catalonia, North-eastern Spain). Lower part of the Perles Formation, Middle Campanian, Upper Cretaceous^35^.

### Diagnosis

Large-sized basal chelonioid defined by the following and unique combination of characters: reduction of the costal plates ossification without a sutural contact between costals and peripherals; carapacial plate margins (costals 5–8 and neurals 5–8) finely sutured; hexagonal/octagonal neural plates, longer than wide, that prevent the costals 6–7 from contacting one another; posterior costal plates that are rectangular-shaped, much wider than long; oval articular area of the ilium, located near the lateral margin of the right costal 8; H-shaped pelvis; enlarged and flat lateral pubic process; conspicuously ornamented, textured surface surrounding the acetabular region; extremely elongated iliac neck; and the absence of carapacial scute sulci, keels, or ornamentation on the external part of the carapace, and absence of the ilium’s posterior notch. *Leviathanochelys aenigmatica* is further diagnosed by having two autapomorphic characters as follows: accessory process on the anteromedial margin of the pubis; and acetabulum strongly ventrolaterally directed.

### Nomenclatural acts

The electronic version of this article in Portable Document Format (PDF) will represent a published work according to the International Commission on Zoological Nomenclature (ICZN), and hence the new names contained in the electronic version are effectively published under that Code from the electronic edition alone. This published work and the nomenclatural acts it contains have been registered in ZooBank, the online registration system for the ICZN. The ZooBank LSIDs (Life Science Identifiers) can be resolved and the associated information viewed through any standard web browser by appending the LSID to the prefix http://zoobank.org/. The LSID for this publication is: urn:lsid:zoobank.org:pub:8288E740-AE81-4F71-8931-86A730182034. The online version of this work is archived and available from the following digital repositories: PubMed Central and CLOCKSS.

### Description

**Figure 2:**
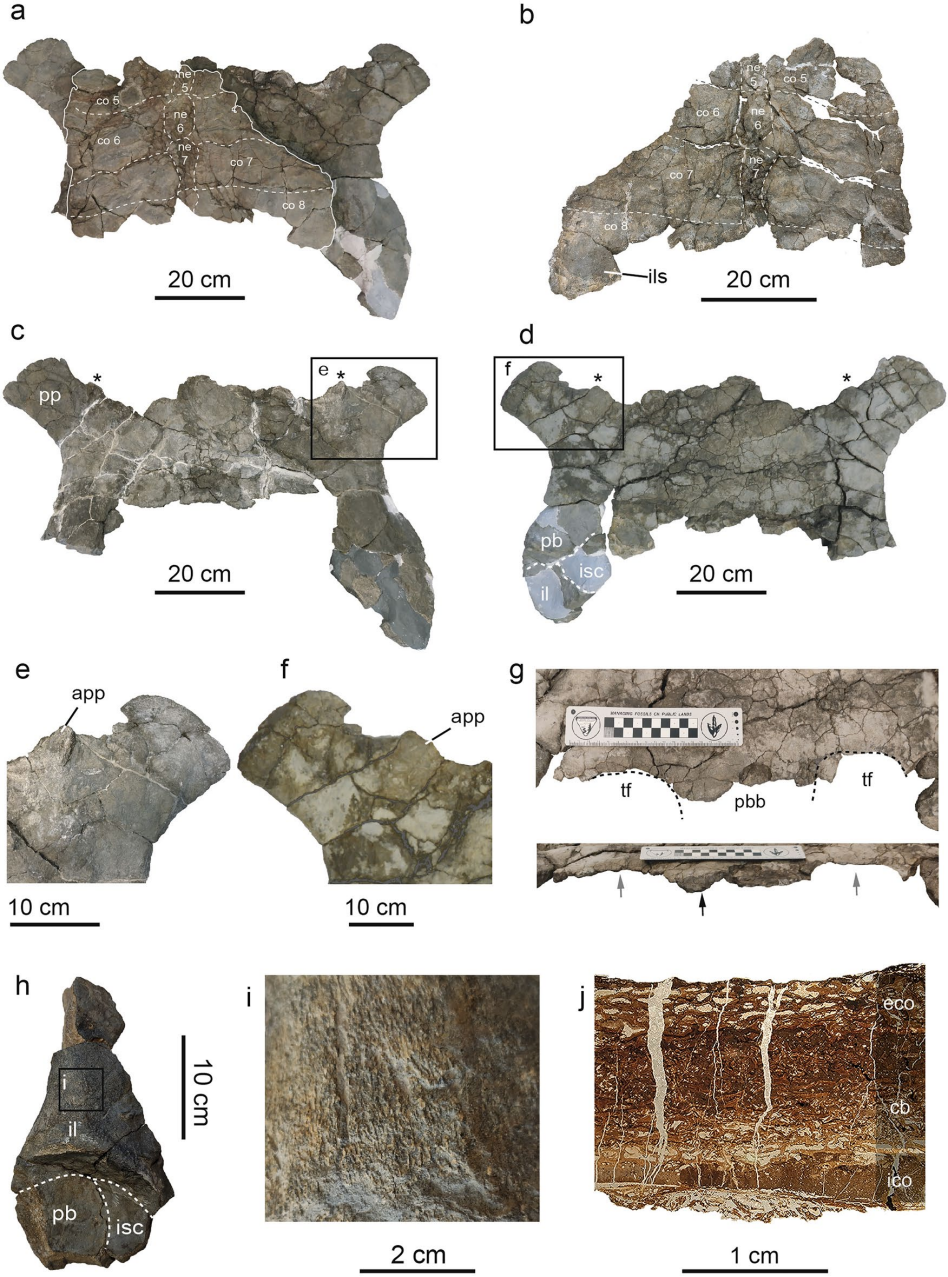
Shell and pelvic girdle elements of *Leviathanochelys aenigmatica* gen. et sp. nov. (**a**) Dorsal view of MCD9884 with the elements disposed as they were discovered, remarking in white the preserved carapace portion (MCD9884a). (**b**) Visceral view of the carapace with a superimposed interpretation of the shell elements. (**c**) Dorsal view of the preserved pelvic girdle without the carapace, and (**d**) ventral view of the same element with the carapace. Asterisk marks indicate the location of the autapomorphic accessory pubic process. Details of the accessory pubic process in (**e**) dorsal and (**f**) ventral view. (**g**) Close up view of the posteromedial part of the pubes, in ventral (upper picture) and posterior view (lower picture), preserving part of the thyroid fossae separated by a thick bone structure (black arrow). (**h**) Ventral view of the left acetabulum, illustrating the limits between the pelvic bones. (**i**) Detail of the outer ornamented surface of the ilium. (**j**) Histological section of the costal 8 (MCD9884.1), showing a cancellous bone zone between the highly vascularized internal and external cortices. Abbreviations: (ac) Acetabulum; (app) Accessory Pubic Process; (cb) cancellous bone; (co) costal plate; (eco) External Cortex; (ico) Internal Cortex; (il) Ilium; (ils) ilium insertion scar; (isc) Ischium; (il) Ilium; (ne) neural plate; (pb) Pubis; (pbb) pubic bridge; (tf) Thyroid fossa.

Only a posterior fragment of the carapace is preserved (Fig. 2a,b), consisting of the remains of the neurals 5–8, and both right and left costals 5–8. The smooth dorsal surface of the carapace is slightly convex, whereas the ventral one is almost flat. No epidermal scute marks are present (Fig. 2a), and there is no evidence of keel or medial shallow depression along the medial axis of the preserved neural or costal plates (Fig. 2a). The left portion of the carapace is the most complete, being their lateral edge slightly sinuous and smooth. The preserved costal plates are subrectangular, much wider mediolaterally than long anteroposteriorly, and finely sutured (Fig. 2a). Their distal edges are slightly sinuous and smooth, which indicates that these plates would have had the lateral extensions rod-shaped to join with the peripheral plates. Viscerally, the right portion of the costal 8 preserves an oval concavity to anchor the ilium to the carapace by ligaments (Fig. 2b, Fig. S1a). The neural series only preserves four elements (Fig. 2a,b): Neural 5 only preserves its posteriormost part, neural 6 is the largest plate and is octagonal-shaped, while neural 7 is hexagonal with short sides in front, and neural 8 is represented by its anteriormost part. It is noteworthy that the neural 7 is highly reduced posteriorly, which would indicate that the total number of elements of the neural series would be equal to or less than nine. In either case, both neurals 6–7 prevent the costals 6–7 from contacting one another along the midline (Fig. 2a,b). Remains of the thoracic vertebrae attachments can be discerned viscerally in both neural plates 6–7 (Fig. 2a,b), highlighting extremely crushed vertebral centra preserved over the neural 7.

As for the preserved pelvic bones (Fig. 2c,d,h, Fig. S1b–f), they form a nearly flat and H-shaped pelvic girdle (Fig. 2c,d). Both pubes are almost complete but lacking most of the anteromedial and posteromedial processes. These bones are flat, smooth, and completely fused to each other. Because of the fragmentary nature of the pelvis, it is not possible to accurately evaluate the expansion of the anteromedial pubic process. The lateral pubic process is flat, square-shaped, and prominent (Fig. 2c–f, Fig. S1b): it extends anterolaterally being deflected about 50º from the sagittal plane of the pubic symphysis (Fig. 2c). A pubic accessory process is located between the lateral and medial pubic processes, which is slightly protruding anteriorly (Fig. 2e,f): it shows a striated pattern on its surface and is slightly convex dorsally and ventrally.

Due to the absence of most of the posterior margins of the pubes, it is not possible to evaluate with confidence if the thyroid fenestra was completely separated (Fig. 2g). However, it is certain that an expanded pubioischiadic bridge would have divided the thyroid fenestrae, at least partially along its medial plane, given that the area for accommodating such process is thicker than the surrounding lateral areas (Fig. 1g). The acetabular contour is oval-shaped and slightly constricted anteroposteriorly (Fig. 2d, Fig. S1b). The acetabulum concavity is completely directed ventrally, and slightly tilted laterally (Fig. 2d). Both the lateral and medial external surfaces surrounding the acetabular region are strongly ornamented with irregular anastomosed ridges. There is no posterior notch in the acetabulum.

Both left and right ilia are partially preserved, but given the fragmentary nature of the right ilium, the following description is mainly based on the best-preserved left ilium (Fig. 2h, Fig. S1c–e). It preserves most of the acetabular region and the iliac neck, which is elongated, and when complete, would have reached an anteroposterior length greater than two times the anteroposterior acetabulum’s length. Ventrally, the medial margin of the iliac neck is nearly straight, while the lateral one is convex, proximally straight, and distally deflected posteriorly. The iliac neck slightly bends dorsomedially (Fig. 2h, Fig. S1c–f). Moreover, its medial, lateral and ventral external surfaces are strongly sculptured with anastomosing anteroposteriorly-oriented ridges (Fig. 2i).

Both ischia are poorly preserved. In fact, only fragments of both left and right ischia, which contribute to the posteromedial region of the acetabulum, are available. According to the preserved graphic documents, it can be stated that the ischium contributed significantly to the acetabulum, and that its main body was likely projected medially (Fig. S1f).

### Phylogenetic relationships

**Figure 3:**
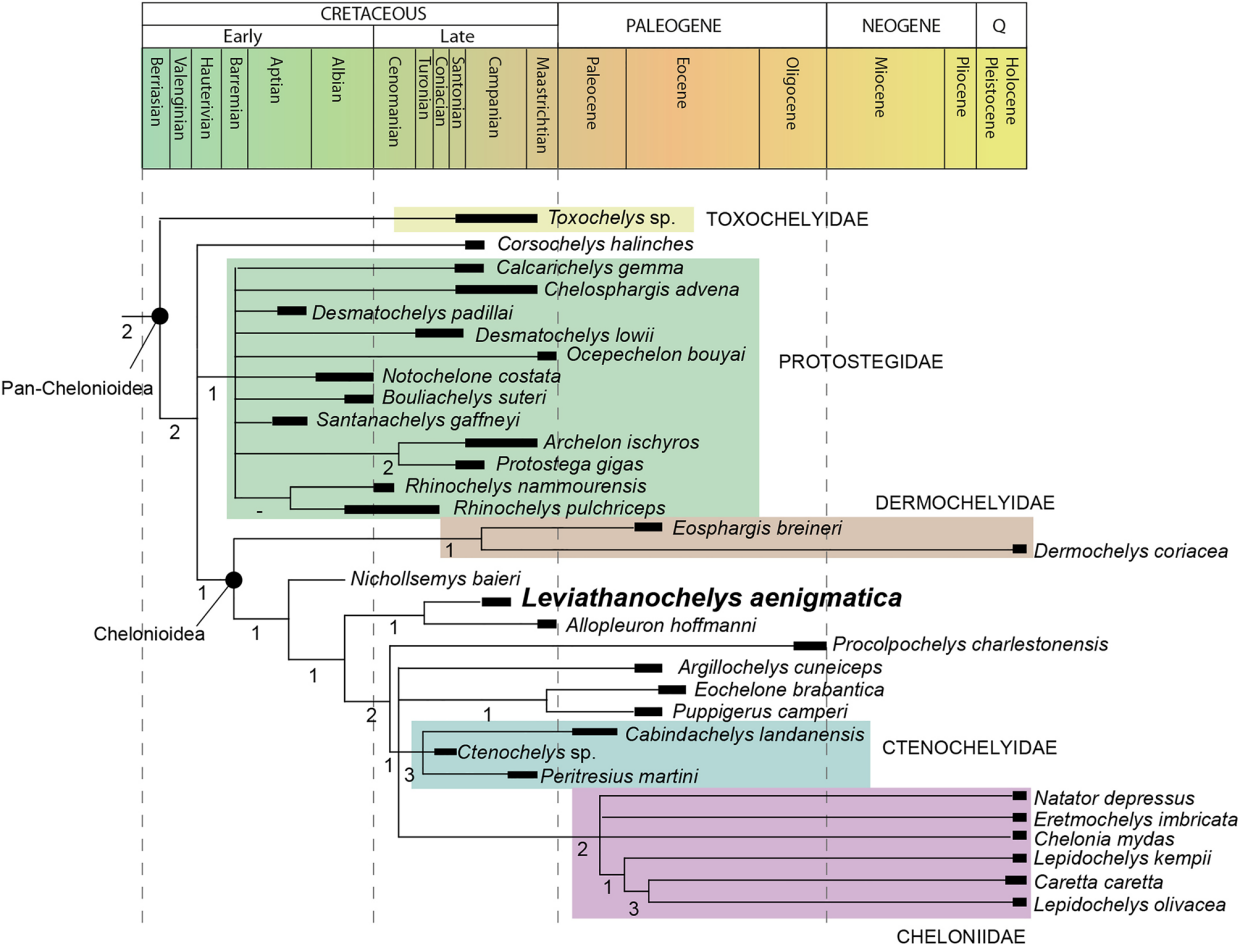
Phylogenetic relationship of *Leviathanochelys aenigmatica* gen. et sp. nov. Simplified phylogenetic hypothesis of the relationship of *Leviathanochelys aenigmatica* within Pan-Chelonioidea based of 20 MPT with 1647 steps according to the Strict Consensus topology. Number under main branching nodes correlate with Bremer support values. Taxa are illustrated according to their time-range occurrence, but not to the time-divergence of the nodes which are tentatively placed according to fossil record evidences.

The parsimony analyses resulted in 20 most parsimonious trees of 1647 steps in length, with a Consistency Index of 0.250 and a Retention Index of 0.686 (Fig. 3). The Strict Consensus topology recovered *Leviathanochelys aenigmatica* as the sister taxon of the basal chelonioid *Allopleuron hoffmanni* (Fig. 3). Nonetheless, it is noteworthy that the only common synapomorphy grouping *Leviathanochelys* and *Allopleuron* is “the attachment of the pelvis to shell by ligaments, instead of a strong sutural contact” (ch. 318:0)^37^. However, this feature is highly plesiomorphic since it represents the typical condition for all Testudinata, to the exception of Pleurodira, Proterochersidae and, maybe also Australochelyidae^38,39^.

The identification of *L. aenigmatica* as a pan-chelonioid is supported by the absence of contact between costal and peripheral plates (ch. 212:1), while the recovery of *Leviathanochelys* and *Allopleuron* as basal members of the Chelonioidea superfamily is established by: the absence of discernible carapacial scutes (ch. 188)^37^; and the presence of a partially or completely separated thyroid fenestra (ch. 319)^37^, a feature that is also shared with *Peritresius martini* and *Erquelinnesia gosseleti* taxa.

Despite being out of the scope of the present study to deeply analyse all the phylogenetic relationships of the recovered topology, it is worth noting that our phylogenetic results located Protostegidae as stem Chelonioidea. As previously mentioned, the phylogenetic position of this group of Cretaceous marine turtles is a matter of an intense debate^5^, nonetheless, our results concur with some of the most recent studies^37^. On the other hand, Ctenochelyidae is recovered as members of Chelonioidea, being its location more inclusive than in other recent phylogenetic studies^5,11^.

## Discussion

### Comparison and taxonomic affinity

The discovery of *Leviathanochelys aenigmatica* in the Middle Campanian marine deposits of the Cal Torrades locality (Southern Pyrenees) represents the first indisputable gigantic bodied chelonioid ever found in Western Europe. The described specimen clearly belongs to a sea turtle characterized by lacking distinct sculpture of the carapace surface, a clear reduction of the ossification of carapace (e.g. costal plates), carapace and pelvis attached to each other by ligaments, and an H-shaped pelvis. Although the entire neural series cannot be evaluated in *L. aenigmatica*, the preserved neural 6 is octagonal-shaped, which differs from the coffin-like neural plates (hexagonal with shorter sides anteriorly) of *Toxochelys latiremis* (taken herein as a representative of Toxochelyidae family^40^; Fig. S2). Moreover, almost all pelvic features exhibited by the genus *Toxochelys* are different from those observed in *Leviathanochelys* (Fig. S3), ruling out any possible relationship between these two taxa.

*Leviathanochelys* shows a conspicuous ornamentation at the external surface surrounding the acetabular region—a bone texture that has been previously correlated with highly vascularized articular surfaces—that is considered characteristic of derived protostegids and dermochelyids^41^. However, this trait might be a plesiomorphic feature, as it seems to be present in *Oertelia gigantea*^42^ as well. Further, the possibility of the new specimen belonging to Dermochelyidae is also discarded, as dermochelyids tend to reduce their shell ossification^3,41^ and, consequently, the size of both neural and costal plates. The described specimen from Cal Torrades has no evidence of epidermal scutes in the preserved neural and costal plates, a condition shared with the protostegids *Archelon*^9^ and *Protostega*^43–45^, and the chelonioid *Allopleuron*^25^, and which further contrasts with the rest of cheloniids. Among protostegids, *L. aenigmatica* resembles both *Archelon* and *Protostega* genera in having a partially divided thyroid fenestrae, as well as an enlarged and flat lateral pubic process; the latter process, which terminates in square-like shape in *Archelon* (Fig. 2e, Fig. S3) and is fan-shaped in *Protostega*^43–45^. Unlike *Protostega* and *Archelon*, the pubes of *L. aenigmatica* are strongly fused to each other; however, this could correspond to an advanced ontogenetic stage, as the degree of pubic fusion changes throughout ontogeny, and reflects different developmental stages of the specimens^46^. Particularly, the extremely elongated iliac neck^9,47^ displayed by *L. aenigmatica* only resembles the condition of *Archelon*. Nonetheless, the absence of continuous or intermittent keels on neurals, and of serrated margins on the carapacial plates^2,44,48^ in *Leviathanochelys* rules out a referral to *Archelon* or *Protostega* genera.

According to our phylogenetic results, *L. aenigmatica* is recovered as a sister taxa of *Allopleuron hoffmanni,* both being considered basal members of the superfamily Chelonioidea (Fig. 3). However, the two taxa differ from each other in several carapace and pelvic features. Firstly, the lateral pubic process of *Leviathanochelys* is much more developed and more laterally directed (about 50° regarding the axial plane) than that of the Centro-European taxon^25^ (35° regarding the axial plane; see Fig. S3). Furthermore, the partially divided thyroid fenestrae of *Leviathanochelys* are smaller than those of *Allopleuron*, and the almost ventrally directed acetabulum of *Leviathanochelys* contrasts with the more ventro-laterally directed one of *Allopleuron*^25^. Finally, the *Leviathanochelys* iliac neck is extremely elongated, with an anteroposterior length at least two times longer than the acetabulum’s length (Fig. S1f), being twice as long as the ratio in *Allopleuron*.

From the aforementioned, it must be highlighted that *Leviathanochelys aenigmatica* has at least two potential autapomorphic features, which have not yet been recognized or mentioned in any extinct or extant chelonioid taxa: acetabulum strongly ventrolaterally directed, and accessory process on the anteromedial margin of the pubis. In summary, based on all the current data available, the new taxon presented here shares multiple features with the members of the superfamily Chelonioidea and the phylogenetic analysis recovers *Allopleuron hoffmanni* as the sister taxa of *Leviathanochelys aenigmatica*. In any case, the identification of *Leviathanochelys aenigmatica* as a new taxa is indisputable, and provides invaluable insight on the evolutionary history of chelonioids as a whole.

### Body size and palaeobiological inferences

*Leviathanochelys aenigmatica* stands out among other pan-chelonioids for its colossal body size proportions, which can be inferred from the large size of its pelvis. The maximum width of the pelvis of *Leviathanochelys* was established at 889 mm (Table S1), which is slightly larger than that of *Archelon* (810 mm wide^45^). The anteroposterior length of the pubis of *Leviathanochelys* (395 mm) is about 119.4% larger than that of *Protostega* (180 mm^44^), and only 14% smaller than that of *Archelon* (460 mm^45^). Despite there not being any allometric correlation between the pelvis size and the total body length in marine turtles, current data suggest that *Leviathanochelys* could be as large as *Archelon*^48^, achieving a body length of up to 3.74 m.

Aside from being the largest marine turtle ever discovered in Europe, and one of the largest worldwide, the finding of *Leviathanochelys* strongly suggests that gigantism in marine turtles was acquired independently, by different lineages over time. The exact evolutionary processes that favoured the acquisition of the larger body size observed in *Leviathanochelys aenigmatica* remain unknown. However, it has been evidenced that the body size of extant marine turtles is related to a combination of environmental factors (i.e. predation pressure, competitive release, temperature) and their ecology (e.g. migration capacity, etc.)^16^.

The identification of *Leviathanochelys* as a marine turtle is further supported on the base of the diploe structures exhibited in the histological sample from the left costal plate 8 (see Supplementary Information; Fig. 2j, Fig. S5), which is characteristic of the main lifestyle of testudines^8,49–51^. The degree of organization and transition between the cancellous bone to the external and internal cortex observed in *Leviathanochelys* somehow resembles that of *Archelon*^49^, suggesting a similar lifestyle for both taxa, i.e. an open water pelagic marine lifestyle (see Supplementary Information).

One of the most remarkable anatomic features of *Leviathanochelys aenigmatica* is the occurrence of an accessory process on the anterior side of the pelvis (Fig. 2c–f). The existence of rugosities and striations around this structure indicate the presence of muscular insertions. Given that this feature is not reported in any other extinct or extant marine turtle, its presence may respond to a very specific function involving the pelvic girdle. Due to its anatomical location and biomechanical function, the pubis is the ankle point of several major muscles involved in the hind-limb mobility and the stabilization of the shell (carapace and plastron^1,52,53^; Fig. S6). Among them, two muscles have their origin in the anterior part of the pubis: the *Musculus rectus abdominis* (RA) and the *M. puboischiofemoralis*^54^ (PIFI and PIFE). RA originates on the anterior part of the lateral process (Fig. S6); it serves as the main stabilizer of the pelvis and acts as a compressor of the plastron during the expiration-inspiration process^54,55^. The *M. puboischiofemoralis* is the largest muscle of the pelvis, and it is divided in the *internus* (PIFI) and the *externus* (PIFE) parts. PIFI is the main extensor of the hind limb, while the PIFE is the main adductor of the posterior limb. Both muscles originate near the pubic symphysis and insert on the minor trochanter of the femur^1,52,54^. Given that the accessory pubic process observed in *Leviathanochelys* projects anteriorly, and has no signs of muscle scar, this suggests backward extension of muscles; it seems unlikely that it would be related to musculature linked to the mobility of the hind limb. On the contrary, the location and the anterior projection of the pubic accessory process suggest that it was somehow related to the plastron and, in fact, it might represent an adaptation related to the respiratory system.

Although there is still some room for speculation, a plausible hypothesis is that the accessory pubic process could have served *Leviathanochelys* as an additional insertion point of the RA (Fig. S6). If so, it could have acted as an additional stabilizer of the pelvis or provided additional compression of the plastron. The morphological features as described strongly support the open water pelagic marine life-style interpretation from histological data (Fig. S5). In summary, anatomical, histological, and myological evidence suggest that *Leviathanochelys* was one of the largest pelagic marine turtles to ever roam the Earth’s oceans.

## Conclusions

To date, it was thought that the largest marine turtles to ever sail the oceans, such as the protostegids *Archelon* and *Protostega*, were restricted to North America during the latest Cretaceous (Campanian–Maastrichtian). The discovery of the new gigantic and bizarre chelonioid *Leviathanochelys aenigmatica* from the Middle Campanian marine deposits of the Southern Pyrenees, which rivals in size to *Archelon*, sheds a light on the diversity of marine turtles and on how the phenomenon of gigantism in these groups was also occurring in Europe. Despite the scarcity and fragmentary nature of the individual, the new evidence not only increases the taxonomic diversity of the Late Cretaceous marine turtle biota in Europe, but also opens a new line of exploration and raises new questions, in order to solve the evolutionary mechanisms and ecological pressures that could have favoured the independent evolution of colossal (> 2.5 m in shell length) marine turtles in multiple lineages, especially during the Late Cretaceous.

## Material and methods

### Nomenclature and terminology

The anatomical shell nomenclature used throughout the descriptions is based on Zangerl^56^.

### Material and institutional abbreviations

The fossil chelonioid remains from Cal Torrades have been recovered through multiple excavations from 2016 to 2021^36^. Because all the remains were found in association to each other and keeping a certain anatomically consistent position, we infer they belong to a single individual (MCD9884), represented by neurals 5–8 (including a highly damaged vertebral centra preserved over the neural 7), costals 5–8 (MCD9884a), and an almost complete pelvic girdle (MCD9884b–g). The pelvic bones include both left (MCD9884b) and right (MCD9884c) pubes, the left (MCD9884d) and right ischium (MCD9884e), and a partially preserved left (MCD9884f) and very fragmented right (MCD9884g) ilia. **MCD** Museu de la Conca Dellà, Isona, Catalonia, Spain; **ICP** Institut Català de Paleontologia Miquel Crusafont, Cerdanyola del Vallès, Catalonia, Spain.

### Osteohistological analyses

Palaeohistological analyses were performed on both the carapace and left ilium. The descriptive terminology used in this study follows Scheyer^57^. Two histological samples were taken from the posterolateral side of the preserved portion of the left costal 8. Following the methodology of Scheyer^57^, the sectioned planes were on lateral and anteroposterior direction, and therefore preserving the anteroposterior carapace plane. Previous to being mechanically sawed, samples were marked to keep the anatomical orientation of the elements. The thin sections were prepared at the Servei de Laboratori de la Universitat Autònoma de Barcelona (UAB). The bone samples were cut with a Buehler Isomet low-speed saw, followed by a polishing process with carborundum powder over a glass sheet. During the polishing process, the grit particles were decreasing progressively in size, on 600, 800 and 1000 grit. Once finished, the next step for the thin-slide preparation, was to fix the sections to a frosted glass slide using ultraviolet curing glue Loctite 358. Posteriorly, the samples were reduced in thickness using a diamond saw (Buehler, PetroThin) of approximately 80 µm. After that, the thin-sections were passed through multiple graded series of alcohol baths and cleared in Histo-Clear II for 5 min. Lastly, the thin-slides were mounted in a DPX mounting medium. They were observed with a Leica DM 2500 P petrographic microscope under both transmitted and polarized light at the ICP facilities.

### Phylogenetic analyses

The phylogenetic analyses performed in the present work are based on the data matrix of Evers et al.^37^, which is one of the most recent, extensive, and updated phylogenetic datasets on marine turtles, including: 96 taxa, and 355 cranial, shell and postcranial characters. *Leviathanochelys aenigmatica* was initially coded in Mesquite 3.04^58^ (See Supplementary File 2), and posteriorly analysed in TNT 1.5 software^59^. All characters were treated as equally weighted and unordered with the new technology search algorithm of TNT, which enabled tree drifting^60^ and parsimony ratchet^61^. The initial level of driven search was set to 30 steps, and the number of times the minimum tree length should be obtained was set to 30 as well. The most parsimonious trees (MPTs) of this analysis were subjected to further tree bisection and reconnection (TBR). TNT was used to calculate absolute Bremer decay indices as a measure of branch support.

## Supplementary Information

Supplementary Information 1.

Supplementary Information 2.

## Data Availability

All data generated or analysed during this study are included in the supplementary information file.
