# Supplementary material for: A gigantic bizarre marine turtle (Testudines: Chelonioidea) from the Middle Campanian (Late Cretaceous) of South-western Europe
Source: Sci Rep. 2022 Nov 17;12:18322. doi: 10.1038/s41598-022-22619-w (PMC9671902; doi:10.1038/s41598-022-22619-w)
Supplement: Supplementary file 1 — Supplementary Information 1. [file 41598_2022_22619_MOESM1_ESM.docx]

**A gigantic bizarre marine turtle (Testudines: Chelonioidea) from the Middle Campanian (Late Cretaceous) of Southwestern Europe.**

Oscar Castillo-Visa, Àngel H. Luján, Àngel Galobart, Albert Sellés

**(SUPPLEMENTARY INFORMATION**)

**
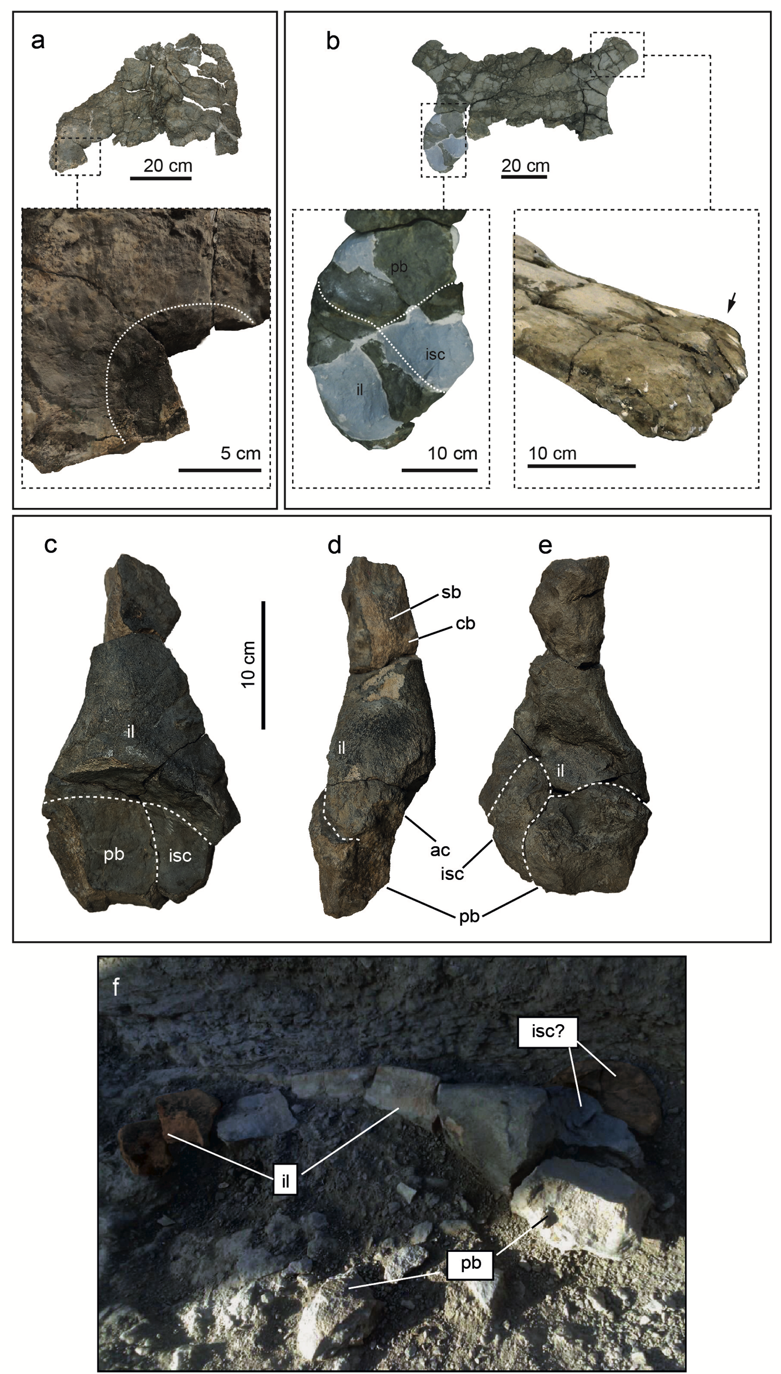
**

**Figure S1.** **Additional shell and pelvic characters of *Leviathanochelys aenigmatica* gen. et sp. nov. (MCD9884a-g) from the Middle Campanian of Cal Torrades (Alt Urgell, Southern Pyrenees).** Detail of the semi-circular iliac scar on the visceral side (a) of the carapace. Pelvis girdle in ventral view (b) showing the anatomic arrangement of the pelvic bones on the ventrally facing acetabulum, and a detail of the morphology of the distal margin of the lateral pubic process, the origin point of the *Musculus* *rectus abdominis* (black arrow). Left ilium and acetabulum in ventral (c), lateral (d), and dorsal views (e). Field picture (f) taken during the original discovery of the isolated left ilium in 2016. Current missing bone fragments are indicated in pale orange, which include part of the ischium. Abbreviations: ac, acetabulum; cb, cortical bone; il, ilium; isc, ischium; pb, pubis; sb, spongy bone.


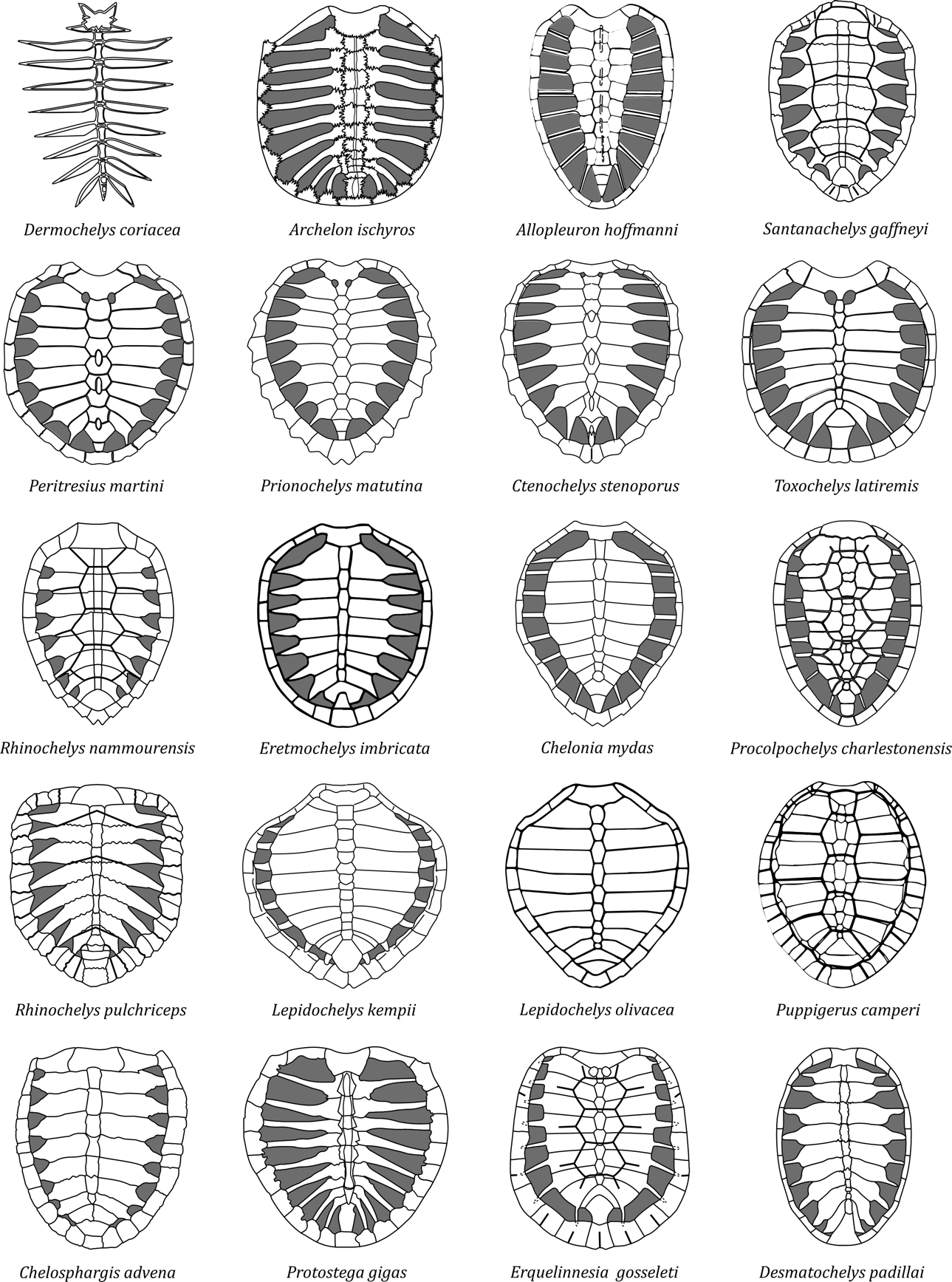


**Figure S2.** **Shell reconstruction of selected Pan-chelonioid taxa**. Schemes of the carapace are not at scale. Schematic shell draws modified from literature^1-17^.


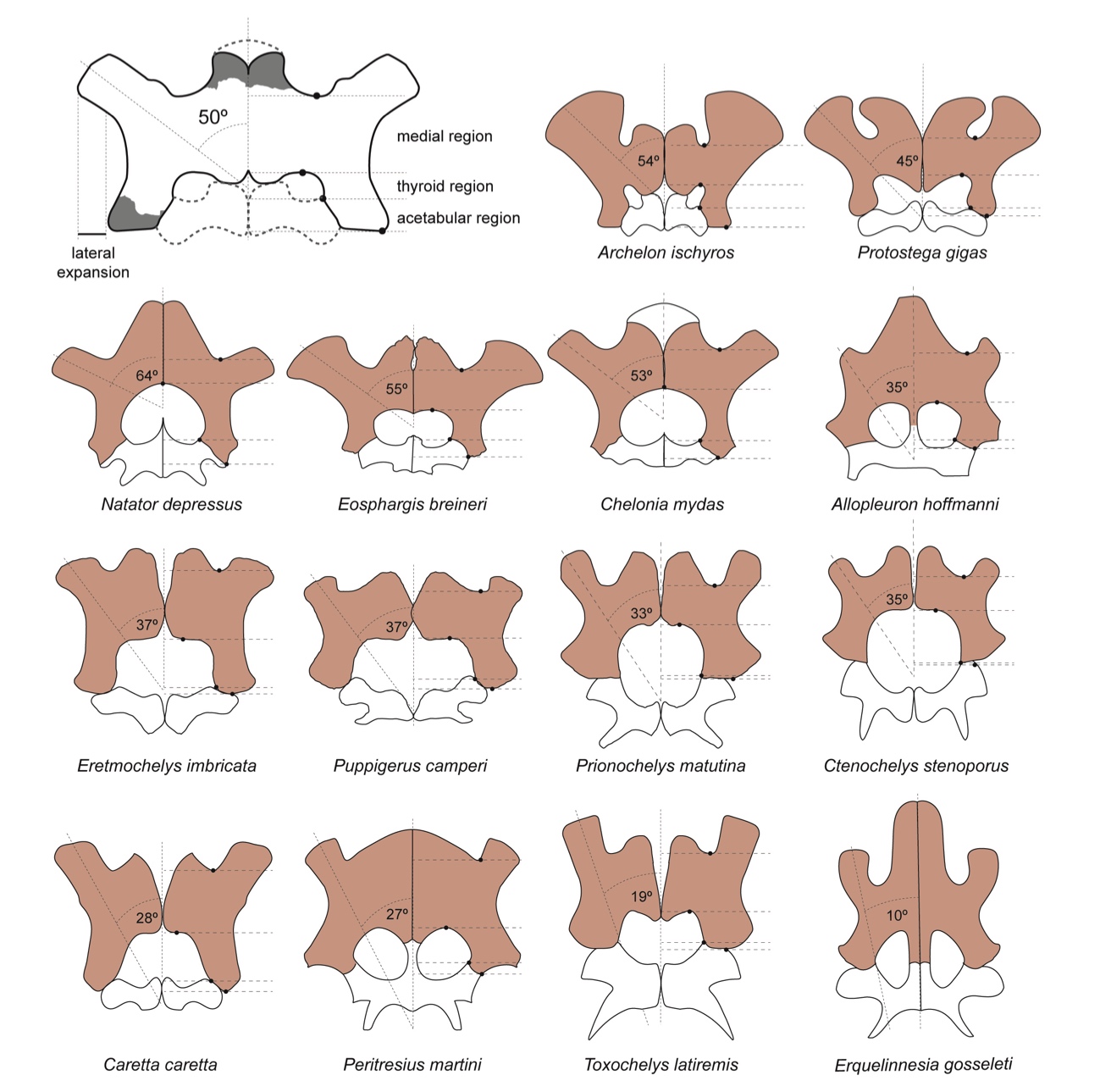
**Figure S3.** **Pelvic reconstruction of selected Panchelonioid taxa.** Schemes of the pelvic girdle are not at scales. Schematic shell draws modified from literature^3, 6, 13, 16, 18-23^.

**2. Supplementary Phylogenetic results**

Leaving aside the phylogenetic position of *Leviathanochelys aenigmatica*, the recovered topology of the Chelonioidea superfamily remains similar to general results obtained by previous authors. As in previous studies^17, 24^, the genus *Toxochelys* was identified as a steam chelonioid. In contrast to the phylogenetic results of Evers et al.^25^, our results recovered Angolachelonia as the sister clade of Pan-Chelonioidea. Such phylogenetic relationship seems to be consequence of the extensive coded missing data of *Leviathanochelys aenigmatica* (95.7%), given that the exclusion of the studied specimen from the analyses returns a Consensus topology similar to that of Evers et al.^25^. However, the obtained topology with the inclusion of Angolachelonia near Pan-Chelonioidea is similar to that of previous studies^5^.

Apart of the abovementioned results, some noticeable differences were observed between our results and the study conducted by Evers et al.^25^ regarding the Strict Consensus topology and the phylogenetic relationships between basal chelonioid taxa (see Fig. S5). For instance, *Corsochelys halinches* was recovered in Evers et al.^25^ as a part of the crown-group Chelonioidea but here is placed as a basal chelonioid (Figs 3, S5A). When visualizing the 50% Majority Rule topology (Fig S5B), it is worth noting the fact most of the tree remains the equal as the Strict Consensus, only improving the resolution of some cryptodirans groups; establishing *Lissemys punctata* as the sister taxa of the group *Petrochelys kyrgyzensis* + *Pelodiscus sinensis* + *Apalone spinifera*; showing affinities between sister taxa inside Protostegidae; splitting and disintegrating the Ctenochelyidae family; and developing the topology of the Cheloniidae family *sensu* Joyce et al. ^26^. In any case, it is far from the scope of this study to take a deep comprehension of these and other phylogenetic differences.

**Figure S4.** **Phylogenetic results**. The main phylogenetic hypotheses of the relationship of *Leviathanochelys aenigmatica* based of 20 MPT with 1647 steps, with a CI value of 0.250 and a RI value of 0.686. Strict consensus tree (a) showing the Bremer Support values below the main dichotomy nodes. 50% Majority rule topology (b) with percentage support below the nodes; values below 50 are not shown. While most of the topology remains stable, the phylogenetic relationships within the Protostegidae and Cheloniidae families are slightly more resolved in the 50% Majority tree.

**3. List of character state synapomorphies**

The following document was created based on the phylogenetic results recovered in our study. Given that the character-matrix used in the present work is based on Evers et al.^25^, ch. 1 to ch. 187 are referred to cranial features, while ch. 188 to ch. 355 are for postcranial and other treats. Here we only provide information of the main testudinate groups involved in the phylogenetic identification of *Leviathanochelys aenigmatica.* Synapomorphies that defines each group and that have been coded in *L. aenigmatica* are highlighted in **bold lettering**.

- Pan-Chelonioidea

39 (0), 75 (2), 211 (01), **212 (1),** 222 (1), 239 (0), 240 (0), 245 (1), 248 (1), 251 (0), 260 (0), 331 (0), 336 (0), 337 (1), 341 (0), 349 (0)

- Toxochelyidae

15 (1), 18 (0), 38 (0), 136 (0), 145 (1), 173 (?1), 199 (0), 274 (?0), 276 (0), 277 (1), 305 (?=1)

- Protostegidae

3 (1), 62 (1), 104 (0), 221 (0), 248 (1), 338 (1)

- Crown-Chelonioidea

145 (1), 261 (1), 291 (0), 292 (0), 329 (1), 346 (0), 347 (1)

- Crown-Chelonioidea (excluding Dermochelyidae)

145 (1), 261 (1), 291 (0), 292 (0), 329 (1), 346 (0), 347 (1), 103 (0), 110 (0), 162 (1)

- Dermochelyidae

11 (1), 16 (0), 53 (0), 65 (0), 87 (1), 99 (0), 111 (0), 150 (1), 293 (1), 331 (1), 332 (3)

- Cheloniidae

5 (0), 151 (0), 301 (1)

- *Leviathanochelys* *aenigmatica* + *Allopleuron hoffmanni*

**318 (0)**

*Leviathanochelys aenigmatica* codding:

Ch 188 🡪 1

Ch 189 🡪 -

Ch 190 🡪 0

Ch 191 🡪 0

Ch 192 🡪 0

Ch 193 🡪 -

Ch 194 🡪 0

Ch 202 🡪 1

Ch 203 🡪 0

Ch 204 🡪 1

Ch 210 🡪 0

Ch 212 🡪 1

Ch 215 🡪 0

Ch 318 🡪 0

Ch 319 🡪 1

Ch 320 🡪 1

Ch 323 🡪 0

Ch 325 🡪 1

**4. Additional histological description**

Two histological thin-sections from the posterolateral margin of the left costal VIII (MCD9884.1-2) and three of the left ilium (MCD9884.3-5) were analysed (Fig. S5). Because carapace samples represent sagittal (MCD9884.1) and transverse (MCD9884.2) sections of the same plates, they display identical osteohistological features. Although the external and internal cortex are partially missing, the characteristic diploe structure displayed by testudines is well preserved, which mainly consist of cancellous spongy bone with large vacuities (Fig. S5a–d, f). The external cortex is more developed and better preserved than the internal cortex, although both are relatively thin in contrast to the cancellous bone region. Both external and internal cortexes show evidence of intense bone remodelling, with large vacuities and secondary osteons (Fig. S5e). Primary bone tissue is only locally preserved close to the peripheral region of the inner cortex (Fig. S5f). It is characterized by showing high-organized parallel fibered bone and longitudinally arranged vacuities, which resembles the lamellar bone tissue described by Scheyer et al.^27^. No LAGs (i.e. Lines of Arrested Growth) are recognized.

As for the ilium histological thin section (MCD9884.3; Fig. S5c, g), it is characterized by displaying a thicker cortex than the carapace sections, and a well-developed medullary region. This latter region of the bone shows large vacuities (Fig. S5g) that progressively reduce their size towards to the cortex (Fig. S5c). The boundary between the medullary region and the cortex is not well-defined, although it apparently consists of a reduction of the vacuities size. The cortex is composed of at least three generations of secondary osteons, which reach the most external part of the periosteal bone. The shape and arrangement of those secondary osteons somehow resemble those described in *Archelon*^28^. No primary tissue has been identified in any of the ilium samples and no LAGs are recognized, presumably due to the great bone remodelling by secondary osteons.


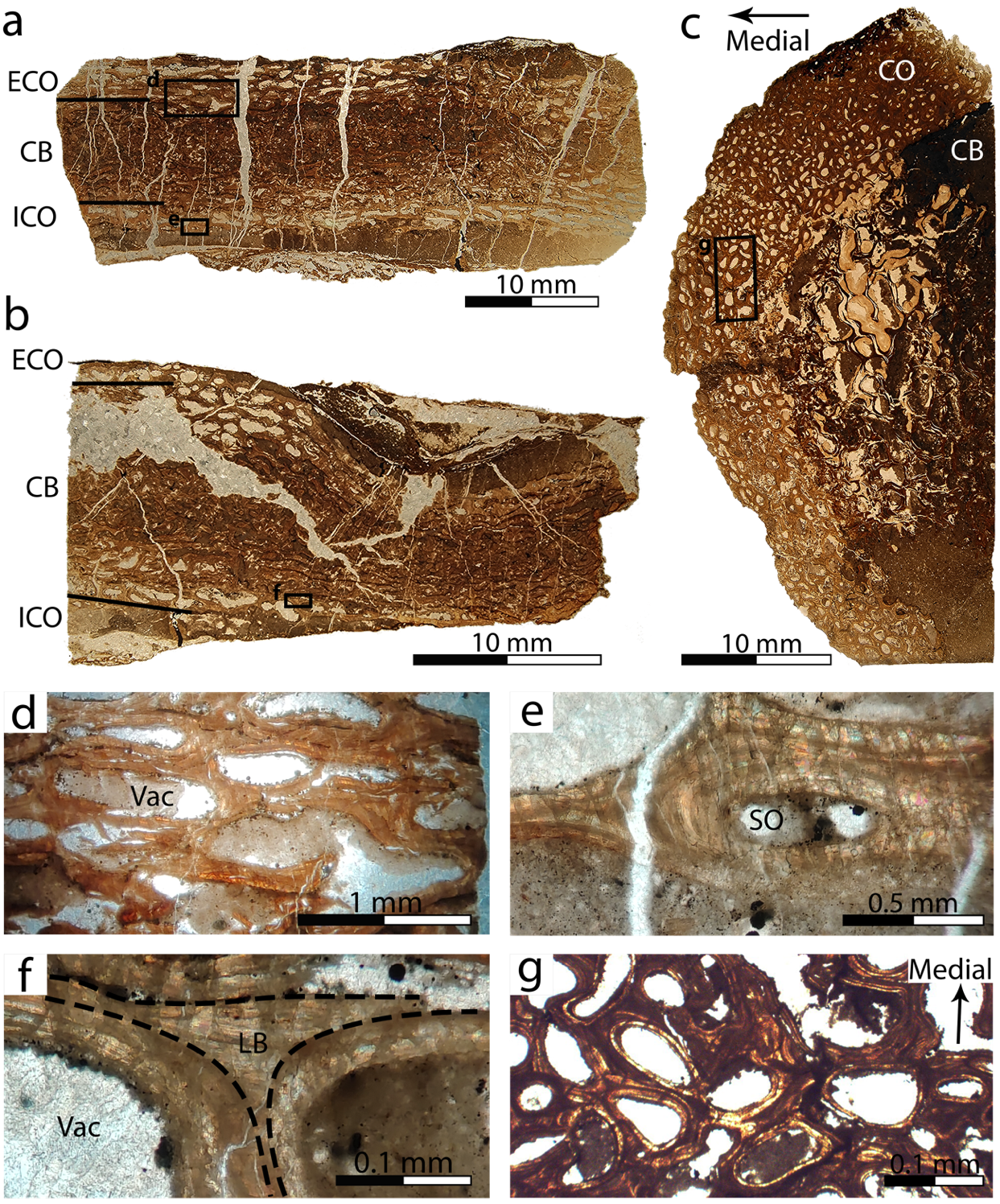


**Figure S5.** **Osteohistology of *Leviathanochelys aenigmatica* gen. et sp. nov.** General view of the (a) MCD9884.1 and (b) MCD9884.2 thin-slides from the left costal 8 plate, and the (c) MCD9884.3 thin-slide from the left ilium, showing the general distribution of the inner bone. Black lines tentatively mark the limit between regions. Detail of large vacuities (d) located near transitional zone between CB and ECO. Detail of a well-developed secondary osteon (e) in the ICO. Remains of the primary tissue (f) between large vacuities close to the inner cortex. Detailed of the ilium vacuities (g) showing a disorganized distribution pattern. Abbreviations: CB, cancellous bone; ECO, external cortex; ICO, internal cortex, LB, lamellar bone tissue, SO, secondary osteons; Vac, vacuity.

**6. Ontogenetic stage**

Despite the absence of LAG’s on the studied samples, the ontogenetic stage of *Leviathanochelys aenigmatica* can be inferred on the base of some osteohistological features. The almost complete obliteration of primary bone tissue by the presence of abundant secondary osteons in both samples from the carapace and the ilium, the expansion of the medullary cavity of the ilium, and the great degree of fusion observed between pubic bones and between carapace plates evidence the advanced ontogenetic stage of the studied specimen at death.

However, and in contrast to other vertebrates, the presence of primary bone tissue in the costal plate, although minority, does not necessarily rule out the possibility that *Leviathanochelys* achieved a senile age at the time of its death. It is show that senil specimens of *Stupendemys geographicus* and *Proterochersis porebensis* still preserve abundant primary bone tissue in their costal plates.

**7. Additional paleobiological inferences**

From an osteohistological perspective, testudinids tend to show a marked differentiation between the medullary zone and the compact bone of the cortex, whereas aquatic or marine testudines display a more diffused and transitional zone between those regions of the bone^28-29^. In addition, the latter show a reduction of the cortex along with an increase of bone vascularization^28-29^. In *Leviathanochelys,* the histology of carapace is characterized by showing a diffuse transition between the cancellous tissue of the medullary zone and the compact bone of the cortex, while also displaying abundant vacuities (some reaching up to 1 mm in length, see Fig. S5a–b, d). These observations are in concordance with a marine life-style.

Despite histological studies have traditionally been focussed in the shell^28-29^, here we also analysed a postcranial bone (ilium neck; MCD9884.3). Overall, the histological observations of the ilium are in the line with what it is displayed in the shell elements (Fig. S7c, g), given that both elements have the presence of a transitional zone between medullary region and the cortex, together with the presence of numerous and well-developed vacuities.

At this point, it is worth noting that the histological characteristics described for *Leviathanochelys* are similar to that of the protostegid *Archelon* (YPM 1783^28^), although some differences are observed. For instance, despite both taxa show a transition between the inner cancellous bones and the compact outer cortex, the boundary is more poorly defined in *Leviathanochelys* than in *Archelon.* In part, this is because the small, dorsoventrally developed vacuities of *Archelon* are somehow more organized than the large, laterally expanded, and randomly distributed vacuities of *Leviathanochelys.* Similarly, the external cortex of the carapace plates of *Protostega gigas* displays a more organized and compact bone tissue than in *Leviathanochelys*. However, both *Archelon* and *Protostega* have a rough shell surface^29^ produced by vascular canals and small foramina, a condition not observed in *Leviathanochelys*.

Houssaye^30^ postulated that the increase in vascularity on both shell and long bones in *Dermochelys* and *Archelon* might have been linked to a fast growth rate, a characteristic that seems exclusive of the gigantic marine taxa, and that it could provide potential advantage in pelagic lifestyle. According to the aforementioned interpretations, the histological features displayed by *Leviathanochelys* are also consistent with a fast-growing gigantic taxon with pelagic lifestyle.

In view of the previous results, the histological differences between *Leviathanochelys* and its alleged sister taxon *Allopleuron* are even more evident. For instance, the external cortex of *Allopleuron* is mainly composed of primary lamellar tissue, only remodelled by few secondary osteons, and it has multiple LAG's^29^. In contrast, the carapace elements of *Leviathanochelys* exhibit numerous large vacuities and some secondary osteons, and neither LAG nor Sharpey’s fibres have been observed. Although those differences might be explained by different ontogenetic stages at the time of death of *Leviathanochelys* and *Allopleuron*, histological samples from *Allopleuron* were taken from the largest individuals, suggesting a real difference in growth rates and lifestyle strategies.


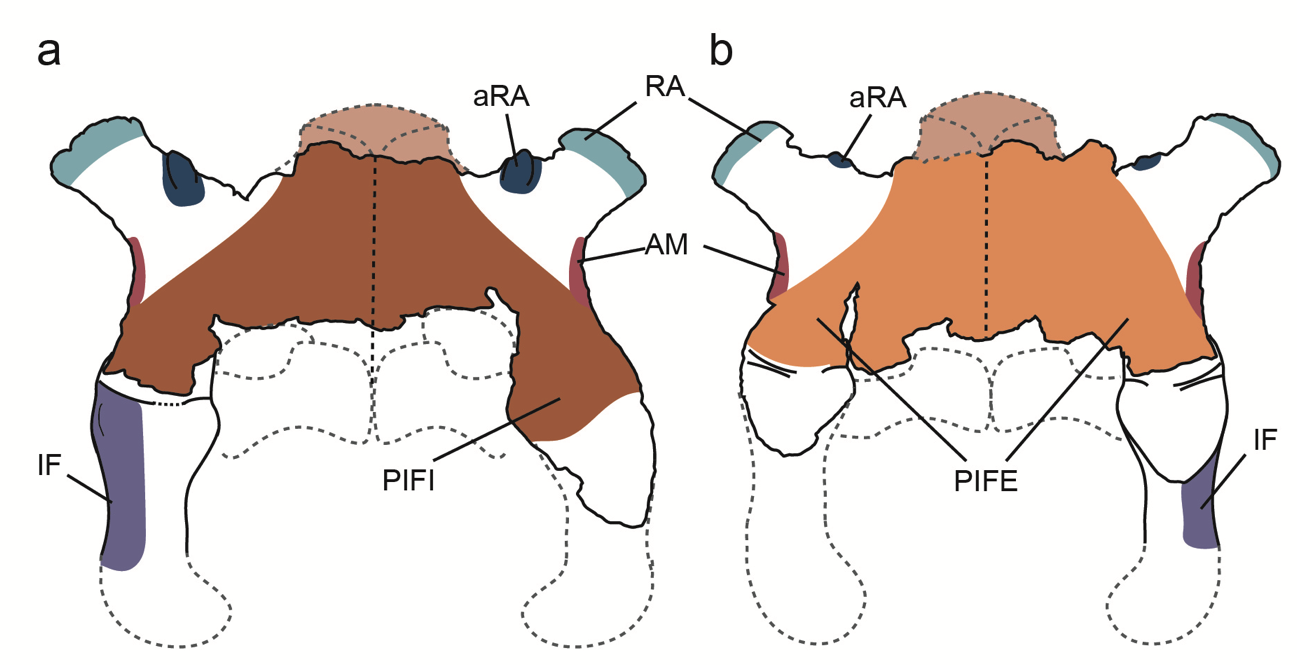


**Figure S6.** **Myological reconstruction of *Leviathanochelys aenigmatica* gen. et sp. nov. pelvic region.** Interpretative myologic map of the main muscles attached to the pelvic girdle of *Leviathanochelys aenigmatica* (MCD9884) in (a) dorsal and (b) ventral view. Abbreviations: AM, *Musculus ambiens*; ARA, Accessory *rectus abdominis*; IF, *M. iliofemoralis*; PIFE, *M. puboischiofemoralis* *externus*; PIFI, *M. puboischiofemoralis internus*; RA, *rectus abdominis*.

**8. Supplementary Measures and Tables**


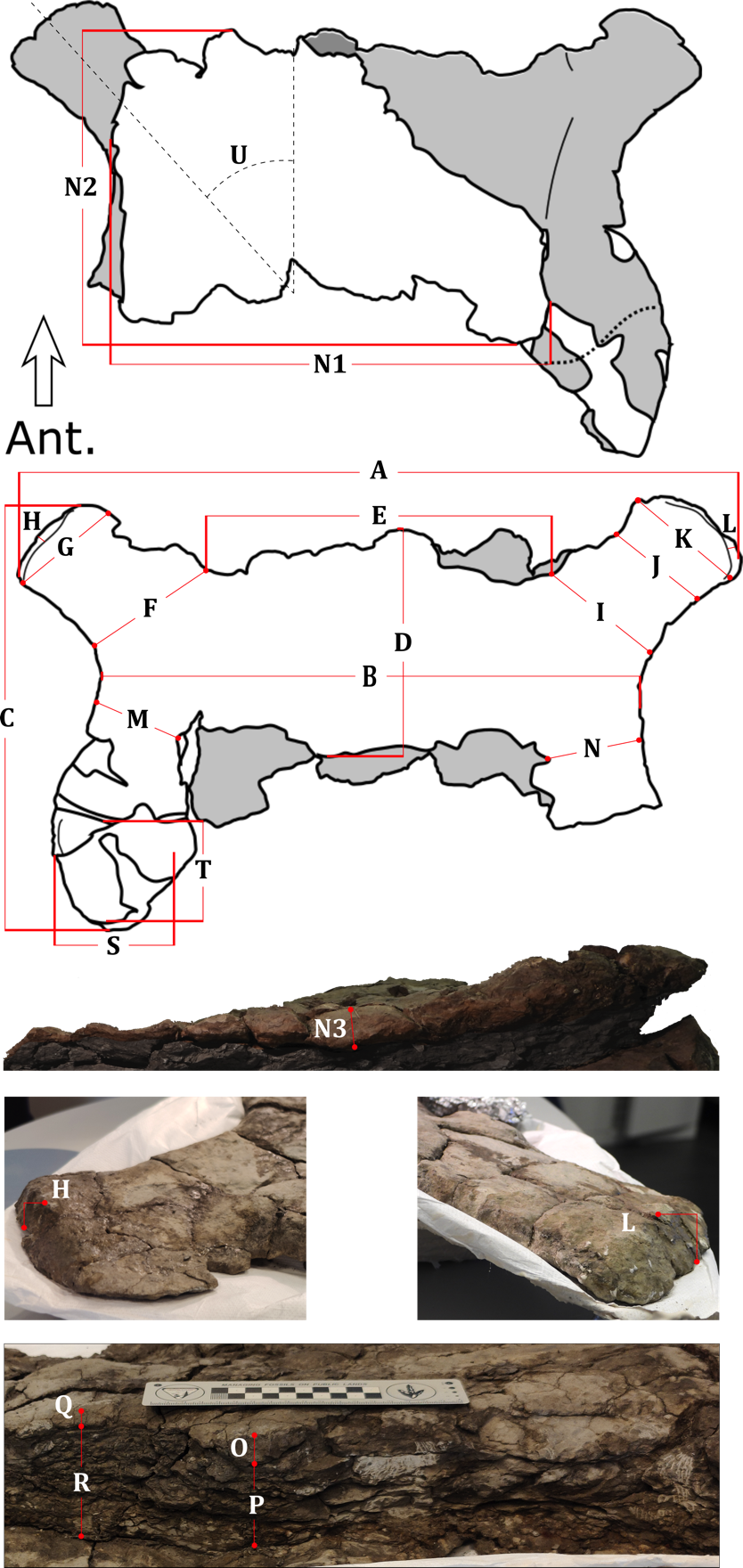


**Figure S7.** **Skeletal lineal measurements of *Leviathanochelys aenigmatica* gen. et sp. nov. with both carapace and pelvic elements attached together.** Letter associated with each measurement correspond to those in Table S1. Scheme of MCD9884, in dorsal and ventral views.

**Table S1.** **Measures of *Leviathanochelys aenigmatica* (MCD9884) gen. et sp. nov.** Based on the illustrated points expressed in the Figure S8.

| **Carapace** | **Code** | **Length (mm)** |
| --- | --- | --- |
| Preserved lateral length | N1 | 542 |
| Preserved anteroposterior length | N2 | 388 |
| Mean preserved thickness on margins | N3 | 14 |
| **Pelvis** |  |  |
| Preserved lateral length | A | 889 |
| Preserved lateral length without lateral and acetabular pubic processes | B | 670 |
| Preserved anteroposterior length | C | 585 |
| Preserved anteroposterior length of the pelvis medial zone | D | 280 |
| Preserved length between medial bases of left and right lateral pubic processes | E | 443 |
| Left lateral pubic process; preserved width on base | F | 171 |
| Left lateral pubic process; preserved width on end | G | 149 |
| Left lateral pubic process; preserved maximum width of the most inclined anterolateral face | H | 20 |
| Right lateral pubic process; preserved width on base | I | 150 |
| Right lateral pubic process; minimum preserved width | J | 127 |
| Right lateral pubic process; preserved width on end | K | 150 |
| Right lateral pubic process; preserved maximum width of the most inclined anterolateral face | L | 30 |
| Left acetabular pubic branch; minimum preserved width | M | 125 |
| Right acetabular pubic branch; minimum preserved width | N | 130 |
| Preserved bone dorsoventral length of the medial part that divides the thyroid fenestra | O | 19 |
| Preserved separation between shell and pelvis on the medial part that divides the thyroid fenestra | P | 46 |
| Preserved bone dorsoventral length of the left thyroid fenestra | Q | 10 |
| Preserved separation between shell and pelvis on the left thyroid fenestra | R | 63 |
| Preserved lateral length of the acetabulum contour | S | 155 |
| Preserved anteroposterior length of the acetabulum contour | T | 140-150 |
| Angle of expansion of the lateral pubic process | U | 50º |

SUPPLEMENTARY REFERENCES

1. Yudha, D. S., Aritona, F. T. S. & Eprilurahman, R. Characteristics of Shell Bone as an Identification Tool for Turtle Species (Reptiles: Testudines) in Java, Borneo, and Sumatra. *Journal of Tropical Biodiversity and Biotechnology*. **5**, 35–43 (2020).
2. Wieland, G. R. Revision of the Protostegidae. *American Journal of Science*. **27**, 101–130 (1909).
3. Mulder, E. Comparative osteology, palaeoecology and systematics of the Late Cretaceous turtle *Allopleuron hofmanni* (Gray 1831) from the Maastrichtian type area. *Pubicaties van het Natuurhistorisch Genootschap in Limburg*. **1**, 23–92 (2003).
4. Hirayama, R. Oldest known sea turtle. *Nature*. **392**, 705-708 (1998).
5. Gentry, A. D., Parham, J. F., Ehret, D. J. & Ebersole, J. A. A new species of *Peritresius* Leidy, 1856 (Testudines: Pan-Cheloniidae) from the Late Cretaceous (Campanian) of Alabama, USA, and the occurrence of the genus within the Mississippi Embayment of North America. *PloS one*. **13**, e0195651 (2018).
6. Gentry, A. D. *Prionochelys matutina* Zangerl, 1953 (Testudines: Pan-Cheloniidae) from the Late Cretaceous of the United States and the evolution of epithecal ossifications in marine turtles. *PeerJ*. **6**, e5876 (2018).
7. Matzke, A. T. An almost complete juvenile specimen of the cheloniid turtle *Ctenochelys stenoporus* (Hay, 1905) from the Upper Cretaceous Niobrara Formation of Kansas, USA. *Palaeontology*. **50**, 669–691 (2007).
8. Gentry, A. D. & Ebersole, J. A. The first report of *Toxochelys latiremis* Cope, 1873 (Testudines: Panchelonioidea) from the early Campanian of Alabama, USA. *PaleoBios*. **35**, 1–10 (2018).
9. Tong, H., Hirayama, R., Makhoul, E. & Escuillié, F. *Rhinochelys* (Chelonioidea: Protostegidae) from the Late Cretaceous (Cenomanian) of Nammoura, Lebanon. *Atti della Società italiana di scienze naturali e del museo civico di storia naturale di Milano*. **147**, 113–38 (2006).
10. Wyneken, J. The anatomy of sea turtles. *U.S. Department of Commerce. NOAA Technical Memorandum NMFS-SEFSC-470*. 1–172 (2001).
11. Weems, R. E. & Brown, K. M. More-complete remains of *Procolpochelys charlestonensis* (Oligocene, South Carolina), an occurrence of *Euclastes* (Upper Eocene, South Carolina), and their bearing on Cenozoic pancheloniid sea turtle distribution and phylogeny. *Journal of Paleontology*. **91**, 1228–1243 (2017).
12. Karl, H.-V., Nyhuis, C. J. & Schollmann, L. The first shell remains of *Rhinochelys pulchriceps* (Owen, 1851) from the Upper Cretaceous of NW-Germany (Testudines: Protostegidae). *Studia Palaeocheloniologica*. **4**, 143–151 (2012).
13. Moody, R. T. J. The taxonomy and morphology of *Puppigerus camperi* (Gray), an Eocene sea-turtle from northern Europe. (ed. Moody, R. T. J.) 155–186 (*Bulletin of the British Museum Natural History*), 1974).
14. Everhart, M. *Protostega gigas dig: October 21 and 23, 2011* <http://oceansofkansas.com/ProtostegaDig.html> (2012).
15. Sett, J. & Maltese, A. *Triebold Paleontology Inc.* <https://www.trieboldpaleontology.com/marine-reptiles/chelosphargis-advena-adult-amp-juvenile>.
16. Zangerl, R. Two toxochelyid sea turtles from the Landenian sands of Erquelinnes (Hainaut) of Belgium. *Memoires de I'Institut Royal des Sciences Naturelles de Belgigue*. **169**, 1–32 (1971).
17. Cadena, E. A. & Parham, J. F. Oldest known marine turtle? A new protostegid from the Lower Cretaceous of Colombia. *PaleoBios*. **32**, 1–42 (2015).
18. Wieland, G. R. The skull, pelvis, and probable relationships of the huge turtles of the genus *Archelon* from the Fort Pierre Cretaceous of South Dakota. *American Journal of Science*. **9**, 237–251 (1900).
19. Wieland, G. R. The osteology of *Protostega*. *Carnegie Institute*. **7**, 279-304 (1906).
20. Nielsen, E. On the post-cranial skeleton of *Eosphargis breineri*. *Dansk Geologisk Forening*. **15**, 281–313 (1963).
21. Zangerl, R. The vertebrate fauna of the Selma Formation of Alabama. Part 3. The turtles of the family Protostegidae. Part 4. The turtles of the family Toxochelyidae. *Fieldiana Geology Memoirs*. **3**, 61–277 (1953).
22. Valente, A. L. S. et al. Computed tomography of the vertebral column and coelomic structures in the normal loggerhead sea turtle (*Caretta caretta*). *The Veterinary Journal*. **174**, 362–370 (2007).
23. Nicholls, E. L., Tokaryk, T. T. & Hills, L. V. Cretaceous marine turtles from the Western Interior Seaway of Canada. *Canadian Journal of Earth Sciences*. **27**, 1288–1298 (1990).
24. Scavezzoni, I. & Fischer, V. *Rhinochelys amaberti* Moret (1935), a protostegid turtle from the Early Cretaceous of France. *PeerJ*. **6**, e4594 (2018).
25. Evers, S. W., Barrett, P. M. & Benson, R. B. J. Anatomy of *Rhinochelys* *pulchriceps* (Protostegidae) and marine adaptation during the early evolution of chelonioids. *PeerJ*. **5**, e6811 (2019).
26. Joyce et al. A nomenclature for fossil and living turtles using phylogenetically defined clade names. *Swiss Journal of Palaeontology*. **141,** 5 (2021).
27. Scheyer, T. M., Syromyatnikova, E. V. & Danilov, I. G. Turtle shell bone and osteoderm histology of Mesozoic and Cenozoic stem-trionychian Adocidae and Nanhsiungchelyidae (Cryptodira: Adocusia) from Central Asia, Mongolia, and North America. *Fossil Record*. **20**, 69–85 (2017).
28. Scheyer, T. M. & Sánchez-Villagra, M. R. Carapace bone histology in the giant pleurodiran turtle *Stupendemys geographicus*: phylogeny and function. *Acta Palaeontologica Polonica*. **52**, 137–154 (2007).
29. Scheyer, T. M., Danilov, I. G., Sukhanov, V. B. & Syromyatnikova, E. V. The shell bone histology of fossil and extant marine turtles revisited. *Biological Journal of the Linnean Society*. **112**, 701–718 (2014).
30. Houssaye, A. Bone histology of aquatic reptiles: what does it tell us about secondary adaptation to an aquatic life? *Biological Journal of the Linnean Society*. **108**, 3–21 (2013).
