# Supplementary material for: A gigantic bizarre marine turtle (Testudines: Chelonioidea) from the Middle Campanian (Late Cretaceous) of South-western Europe
Source: Sci Rep. 2022 Nov 17;12:18322. doi: 10.1038/s41598-022-22619-w (PMC9671902; doi:10.1038/s41598-022-22619-w)
Supplement: Supplementary file 2 — Supplementary Information 2. [file 41598_2022_22619_MOESM2_ESM.docx]

#NEXUS

[written Sun Jul 31 10:55:48 CEST 2022 by Mesquite version 3.70 (build 940) at LAPTOP-IFOGLUHU/192.168.1.149]

BEGIN TAXA;

TITLE Taxa;

DIMENSIONS NTAX=97;

TAXLABELS

Rhinochelys_pulchriceps Santanachelys_gaffneyi Bouliachelys_suteri Notochelone_costata Ocepechelon_bouyai Desmatochelys_lowii Desmatochelys_padillai Rhinochelys_nammourensis Chelosphargis_advena Calcarichelys_gemma Protostega_gigas Archelon_ischyros Dermochelys_coriacea Eosphargis_breineri Lepidochelys_olivacea Caretta_caretta Chelonia_mydas Eretmochelys_imbricata Lepidochelys_kempii Natator_depressus Puppigerus_camperi Argillochelys_cuneiceps Allopleuron_hoffmanni Nichollsemys_baieri Eochelone_brabantica Corsochelys_halinches Procolpochelys_charlestonensis Peritresius_martini Ctenochelys_sp Cabindachelys_landanensis Oligochelone_rupelensis Erquelinnesia_gosseleti Toxochelys_sp Solnhofia_parsoni Jurassichelon_oleronensis Portlandemys_mcdowelli Plesiochelys_planiceps Plesiochelys_etalloni Plesiochelys_bigleri Sandownia_harrisi Brachyopsemys_tingitana Leyvachelys_cipadi Angolachelys_mbaxi Chelydra_serpentina Macrochelys_temminckii Sternotherus Staurotypus Kinosternon_suburum_hippocrepis Dermatemys_mawii Baptemys_wyomingensis Emarginachelys_cretacea Chrysemys_picta Emys_orbicularis Geoclemys_hamiltonii Testudo Gopherus_polyphemus Chelonoidis_sp. Platysternon_megacephalum Apalone_spinifera Pelodiscus_sinensis Lissemys_punctata Carettochelys_insculpta Allaeochelys_libyca Chelus_fimbriatus Phrynops_geoffroanus Elseya_dentata Chelodina Araripemys_barretoi Podocnemis Pelomedusa_subrufa Ordosemys_sp._IVPP_V12092 Judithemys_sukhanovi Annemys_levensis Annemys_latiens Annemys_sp._IVPP_V18106 Xinjiangchelys_wusu Xinjiangchelys_radiplicatoides Dracochelys_bicuspis Kirgizemys_hoburensis Kirgizemys_dmitrievi Sinemys_gamera Sinemys_lens Meiolania_planiceps Chubutemys_copelloi Eubaena_cephalica Arundelemys_dardeni Pleurosternon_bullockii Glyptops_plicatulus Kallokibotion_bajazidi Eileanchelys_waldmanni Kayentachelys_aprix Australochelys_africanus Proganochelys_quenstedti Adocus_lineolatus Petrochelys_kyrgyzensis Galianemys_whitei Leviathanochelys_aenigmatica

;

END;

BEGIN CHARACTERS;

TITLE Character_Matrix;

DIMENSIONS NCHAR=356;

FORMAT DATATYPE = STANDARD RESPECTCASE GAP = - MISSING = ? SYMBOLS = " 0 1 2 3 4";

MATRIX

Rhinochelys_pulchriceps 00100000111100211000110?0100??00000000011000011100000000001?0111010100001020(12)10201000011?1211011010011001111200?0000020100000000010202000002200210?121000011000011001?1??????00110(01)1?000000?????????????????????????????????????????????????????????????????????????????????????????????????????????????????????????????????????????????????????????????????????????

Santanachelys_gaffneyi 0010000?101100?11?00110??100??000000000??00001110?00000000??011101010?001021?1020?0?0011?121?010000011001101200100?0???10?????0????2???0000????????1200?0011??????????1??????00???0????????00000?0??0?000101001?00?11100?01000???01?0000?01100?0110112012?01?1??0?0010?0???????????????11???????????0???????1?1????111021????0????0???1?1??2???0101101021010?1?1????

Bouliachelys_suteri 0011000?0010001??000110?0100??000?00?0?1?0?0011100000000001?0111010100001020010201010011?121101011001100111120010000020100010000010202100002210210?121000111000011101?1??????0011011100?001?????????????????????????????????????????????????????????????????????????????????????????????????????????????????????????????????????????????????????????????????????????

Notochelone_costata ????000?1011002??0?0110?0?00??00?000???1?0000111000?0000001??1110100010010???102000?0?11?121?010000011001111200???1???0??0000010010200000?02210210?121000011000011001?1??????0011??????????????????????????????????????????????????????????????????????????????????????????????????????????????????????0????????????????????????????????????????????????????????????

Ocepechelon_bouyai 0000?010101101?0000011???100??000000001?10000111100000?0000?011101000?00?02021020?0?001011210020?100110010?1200000?0120100???????002??00000221?1???1210?0111?000????1?1?????????????????????????????????????????????????????????????????????????????????????????????????????????????????????????????????????????????????????????????????????????????????????????????

Desmatochelys_lowii 00100010101100110001111?0100??00000000010000011101000000001?011101010?001020110201000011?121?000110011001??12001000???0???0?001?00021200000221021??121000111000011101?1??????0001?0???????100000?0100?0101010??001111100?010???01?1??100001110?021011201?101?1??0?011000???????????????1011011120??0010000201??????111021111?0?1??0011101112011011111102?????1?101?1

Desmatochelys_padillai 0010??1?101100?10011?????100??000000000?100001110??000?000??01110???0?0010201102??0?001??121101001002?001111200100?01201000???????02?2?0000????????1210?0011?00011101?1??????00???0???0?00?00000?0100?0001010??00??1110??0?0???01?1????0001????????????????????????????????????????????10110111200000100002?10?????1110211???????????????112011??11?11021????1?????1

Rhinochelys_nammourensis 0010??00111100?10?00?????100??000000000???0001110??0?0????????????????????????????????1??????0?????????????????1000????????????????????????????????????????????????????????????????????????00010?0??0?000101001?00?10?00?00000??????1100?01110?0110112012101?1??0?0010?????????????????????????????????????01????????????????0???????????1?2??1?111111021010?1??????

Chelosphargis_advena 0010??0?1011001110?01???0100??00000000???00?01110?0000??0?1?01???????????0?0?10????0?01??1??1??????????????120??0???020????????????????????????1?0?1210?0011????????1????????0011?????0??010?010????0?0001???01000?11100?01?00???01?110?0?11?000110112012101?1??0?0110???????????????????????????????????????????????????????0??????????????????????????????????????

Calcarichelys_gemma 1??1??0?101?00???0?011??????????00?000?????0?1110?00000???1?(0 1)1??0????????02?21020100001??12110?0?00???0010?1200100?00201000?0?????020??000022??210?1210?0011?0001100??1??????0011??????????0?010??110?000101?01000?11100?01?00?1??1?110?0011?0?0110112112101?1??0?011?0001????0?0000??????????????????????201????????????????0????????1????????????????????????????1

Protostega_gigas 1??0??00101000??0?00?????100??000000?0???00001110?001000001?011100000???102?21?21?0?0?1??1?1102??102?1011??1?0?100?0?0?1000???????02???000?????2???1200?0011?000??????1??????000100??00?0001?010?0100?000101001000?11110?00000?1?11????0??1110?0210112112101?1??0?01101????????????????111?011120????10000201011110111021111?001??00111011130111111111021010010101?1

Archelon_ischyros 1??0??00101000?00?001????100??00000?0001000011110?001000001?011100000????021210???0?0?1011211020?102??011??1200100?0?0?10?0???????02???000???????????????????000??????1??????0001?0???????11?010?0100?000101001000?11110?00000?1?11????0??1110?0210112112101?1??0?01101????????????????101?011?????????0??201?11110111021111?011??00111011130111111111021010010101?1

Dermochelys_coriacea 1??10100101000001?00100?0000??00000000010010011101000100010?0101010000011021210100000011?1210021?002??0000?1200000(0 1)00001030?01?0010202000000210111?1211?0111000011101?1??????0001011??0?2011??0??0110??00?1??1?00??????????0???1??1???????111??0011?????1101?1??0?01111????????????????111101112000110000120?011110111021111?001??0011?010131110100111021001?1?11101

Eosphargis_breineri 1??1??0?101000??1?00?????100??0000000?????0001110?0001?0011?010101010????02??10???000?1??1200020?002??1000?1200000?00?0111????????0????0110????????1211?011?????????1?1????????????????????1?01??0???????00??0?????11111???????????????????11??011?????????????????1111?????????????????11?01????????0?0?????????????????1????01????11?01013111?1???110?1????1?101?1

Lepidochelys_olivacea 1??10101101000101000110?01011000000000010000111100000001110?0101000211011021210201000011?1210020?100201100?1201100(0 1)0000110000010010201001002210210?121010111000011001?1??????0111111100100100010?0110?00010000?000?1110110100110111?1100?11110?0100112011101?1??0?01110001???00(0 1)00000001111011120001110001201111110111020111?001??001110111201111101110111010101110?

Caretta_caretta 1??10101101000101000110?01011000000000010000111100000001110?110110021001102121020100(0 1)011?1210020?101?11100?12011000000011?000011010202001002210211?121010111000011101?1??????0001111100100100010?0110?000101000000?1110110100100111?1100011110?0100112011101?1??0?01110001???(0 1)0(0 1)00000001111011120001110001201111110111020111?001??0011101112011111011101110101011100

Chelonia_mydas 1??1010?101100101000110?010111000000000100001111000000011120010100021000102121010100001(0 1)11210020?102?00000?1201100101001110001110102000010020?0111?121010111000011001?1??????0011110100000100010?0110?0(0 1)0101001000?1110100100110111?1100001110?0100112011101?1??0?01110001???10(0 1)000000011110111200011100(0 1)1201111110111021111?001??0011101112011111011101110101?11100

Eretmochelys_imbricata 1??10101101100101000110?0101100000000001000011110000000111200101000211011021210201000011?1210020?100211100?120110000010111000010010200001002210111?121010111000011001?1??????0000111100000100000?0110?0(0 1)0101001000?1110110100110101?1100001110?0100112011101?1??0?01110001???10(0 1)00000001111011120001110001201111110111021111?001??0011101112011111011101110101011100

Lepidochelys_kempii 1??10101101100101000110?01011100000000010000111100001001112?0101000211011021210201000011?1210020?100211000?1201100(0 1)0000110000011010202001002210110?121010111000011001?1??????0011101100000100000?0110?00010000?000?1110110100110111?1100011110?0100112011101?1??0?01110001???00(0 1)00000001111011120001110001201111110111020111?001??0011101112011111011101110101011100

Natator_depressus 1??10100101100101000110?0101110000000001000011110000000111200101000211011020210101000011?1210020?100211100?120110010000110000010010202001002210211?121010111000011001?1??????0010110100100100000?0110?000101001000?1110110100110101?1100001110?0100112011101?1??0?01110001???10(0 1)00000001111011120001110001201111110111021111?001??0011101112011?1101110111010101111?

Puppigerus_camperi 1??10000101100101000110?11011100000000010000111100000001110?0101000210001021210201000011?1200020?100201100?12011000010011100001?010202001002200110?121100111000011001?1??????0111101100000100000?0??0?000101001000?0??0120100100111?1100001100?0000112011101?1??0?0010000?????????000??1111011120001?0000120???????111021111?001??0010?1111201101?0?1101?????1?1??10

Argillochelys_cuneiceps 1??10000101100101000110?01011100000000010000111100000001111?010100021000102121020100001011210020?1002?1100?120110010100110000010010202001002210111?121100110000011001?1??????0011101100?001???????????????????????????????????????????????????????????0????????????????????????????????1111011120001110001?010??????????????????????????????????????????????????????

Allopleuron_hoffmanni 1??100001011001?0000110?00011?00000000010010011101000001110?010100021?0010211101110010101121000011002?1100?1201???10000110000010000212001002210111?12100011100001100011??????0000???10000001?010?0100?000101001000?1111??0100111?01?1100001110?0100112011101?1??0?01111????????????????1111011120001110001201011110111021111?011??00111010120110110111011101010??1?0

Nichollsemys_baieri 1??1000?1011001?0010110?0101010000100001000001110?000000010?0111000100001021210201000011?021?0000100010110?1201100?0000110001011?00202001002210111?121010111?00011001?1??????0001?????0?00??????????????????????????????????????????????????????????????????????????????????????????????????????????????????????????????????????????????????????????????????????????

Eochelone_brabantica 1??10000101100101000110?11011100000000010000111100000000000?010101011000102121020100001011210020?100211000?12011000010011200001?010200001001200111?121000111000011001?1??????0001101100000100000?0110?0?010?001000?1110120100110111?1100001100?0000112011101?1??0?0110??????????????????11??1??????????0??20???1???111021????0?1???0?????01201101???110?1???01?10110

Corsochelys_halinches ???????????1011000?0111?0??????????0000?0???????????????????????????????????2???????0?1011210??0?10???00?0?1200100100?1???0000001?02?200000121?210?12??00?11100011101?1??????????????????????000?0??0?00??01001??0?11001001?011????????????110?01??1??011101?1??0???????????????????????11?00????????0?00??????????11102111????1??00??????01011????????2????????????

Procolpochelys_charlestonensis 1??1000?101100101?001????10110000000000100001111000000?1110?010100021?0?1021210???00001??12?0020??01?01100?1201100?????11??????????2???010022??????121??0111????????1?1??????0101111?00?00100000?0??0?100100001000?10?01201000???1??11????1110?010011201??01?1??0?0111??????????????????11??1?????01?????1?????1???????2111??????????????0120110???????????????101?0

Peritresius_martini ???????????????????????????????????????????????????????????????????????????????????????????????????????????????????????????????????????????????????????????????????????????????????????????00110?0010?010101001000?????120100110101?1000001110?010??????1101?1??0?011000??????000000?01??1??1???????1???11201????????????????011??001??1???????????????????????????0

Ctenochelys_sp 1??1010?10110111000011??1101110000000001000001110?000001111?010101111?00102?21020100001011210000110001000111201100101001100??00???02???01001210111?121000111?00011111?1??????0011111?00?00100110?0010?010101001000?1110100100110101?1100001110?0100112011101?1??0?01100001????000000?00111??1012000111000120101?????11?21110?001??001??1?1100110110?1101?????1????10

Cabindachelys_landanensis 1??1000?1011011?100012??110111000000?????000011100000001110?01010002100?1021210201000011?121?0000100010000?1201100?0?001100?000?0??????01002200111?12100011100001100??1????????????????????00110?0???????1??????????????????????????????????????????????????????????????????????????????????????????????????????????????????????????????????????????????????????????

Oligochelone_rupelensis ???????????????????????????????????????????????????????????????????????????????????????????????????????????????????????????????????????????????????????????????????????????????????????????00000?0??0?000101001000?11101?0100110111?1100001110?0100112011101?1??0?0111??????????????????1???1??????11??0?1?0???????111021?11?0?1??0010???0120110????????????????????

Erquelinnesia_gosseleti 1??10?0?1011011???001???0?0???00?0??????00?0?1111?0000?1110?01?1000210???021?1020??0001??1???0??????????0????????????????????????????????????????????????????????????????????0101101100000100000?0??0?0(01)0101001000?110012010011??11?10000011?0?010????????01?1??0?011???????????????????1?1?11???0011???01?0?????????????????011??0010???012001?1????????????1?1010?

Toxochelys_sp 0011000?10110111001011???101010000000011?000011101000000011?01010001(01)000102121020000001011210000010001000111200100000001000?000?0?0202001000200111?121000111000011001?1??????1001(01)11100100100000?0110?010101001000?11000?0100110111?11000011(01)0?0100112011101?1??0?001000???1??0???1011011?10111200??0????1?0101??001110?00???00???0???11110200?01101010?0010?1?0?000

Solnhofia_parsoni 0011000?1011011??100111101010100001?00?000?0011100000000100?1111100200001020210201011010112100001001?0001101100???100201110(01)0000110200000000100110?121000111000011001?1??????0111001111101000000?0????0?0101001?00010?01?01002?01?1?0100?01100?100????????1001??10000000??????000?000??110?00????????0?0????1?1????111120????0??????0?1?1?00011?0?01000?0???0000?1?0

Jurassichelon_oleronensis 0010001?10110110001011110100??00001000010000011101000000001?01110000000010212102000110101111001101001100110120100010?00100010001100212000000200110?111000011100011001?1????????????????????0000??0100?0001010???0?????????10???????????0???1?0?100???10???1001????????00??????0?0?00???11??00????????0?0??1???????????????????????????1????????????????????????0?1??

Portlandemys_mcdowelli 0010001?1011011??00011110?00?????0??000100?0011101000000001?010100000000102?2102010110101111000001000100?101201?0010100100010001(01)00212000000200110?121000011000011001?1??????000000?1001010?????????????????????????????????????????????????????????????????????????????????????????????????????????????????????????????????????????????????????????????????????????

Plesiochelys_planiceps ???0000?1011011?00?011110100??0?0?2?00?100?0?11?0100?000001?011100000000102?2102010110101121001000001100110110110010120100000001110202000000210110?121000011100011001?1??????00000011011010?????????????????????????????????????????????????????????????????????????????????????????????????????????????????????????????????????????????????????????????????????????

Plesiochelys_etalloni 0011000?1011011?00?011110100??00001000010000111101000000001?0101000000001020210201011010112?00100000110010?1200??????????0000001110202000000210100?121000011100011001?1??????0000000101101000000?0100?000101001?0100??01?0000100011?0100000(01)00?1000111000?10?1??10000000100100000000000????????????????????010?????1111??????0?1??0?????1??00110?001000?????????????

Plesiochelys_bigleri ?????????????????0?011110100??0000???0010000?1????????000?1??101?????????020210201011010112100100?0011001101100?00100?0100000001110202000000200010?111000011100011001?1????????????????????00000?0??0?00010100100100??0??0000100011?01000?0(01)00?1000111000110?1??1?000000100100000000000????????????????????????????1111?0??????1??0?1??1??000??0??0????????????0?100

Sandownia_harrisi 0111001?1011011?0000121101010100000000?0?000011100000011110?01010002100010202202000110101111?0001101??00110121100000010?0?010001010201000?00000010?111000111000011001?1??????01010??1111011?????????????????????????????????????????????????????????????????????????????????????????????????????????????????????????????????????????????????????????????????????????

Brachyopsemys_tingitana 0111000?10110110100011??0101010000000000?000011110000000110?010110021001102?21020001101??1?1?0000101?10011112111000010010?0?0?????0201????0?2??0?0?121000111?0001110011??????010100?1111011?????????????????????????????????????????????????????????????????????????????????????????????????????????????????????????????????????????????????????????????????????????

Leyvachelys_cipadi 0001??1?10110110100012???100??00000000001000011101000000110?0111000?1?0?102022020?01101011?1?0000?01??001111211100?00?0?010???????0??1????000011?0?1210?0111?000??????1??????01???0?????0?1?????00??0??0????001?0??0??00?0???????1??1?????0?????????????????????1??????????????????????111?01????????1?0??201??????????2?0???0????0?0????1000?1?0???000????????????0

Angolachelys_mbaxi ???1000?101101?0100012???101110000000010?00001101?0000?1110??101?0021????02?22020?011010?1?10020??01?1?0?0?1210000?0?1010?????????02?2??000????????1210?0111?00011001?1??????0101??????????????????????????????????????????????????????????????????????????????????????????????????????????????????????????????????????????????????????????????????????????????????0

Chelydra_serpentina 1??10000001000110020111011010100000000001000011101001000001?01010100010010212202010000101121?00011000100010120010(01)10100100001011010200000000101010?12110011000001100011??????0011011000000100000?01012000001001000?10?00?01002?0101?1100001100??000112012101?1??0?01100001?1?0010?011001111011120010111010201111200111020011?001??00011?1100001?00010001001????????0

Macrochelys_temminckii 1??10000101000110020111001011100000000000000011101001000000?01010000010010212202010010101121?00011000100010120010(01)101101000000110102000000000?0110?12100011100001100011??????0011011000000100000?01011000001001000?10?00?01000?010001100001100?1000112012101?1??0101100001?1?00?00011001111011120010111010201112200111020011?001??00001111000?1?00010001001?10?????0

Sternotherus

1??10000101000110120120?11(01)11100100100001000011101000010000?01010102110010212202000000101121?0011001?100010120010000000102001001000210000010100010?12110011000101100011??????000000?01001110011010101100010100211110??00?01000?0111?110000000111001?????010021??0100000001?1?11?1?1?001111101111001011101021?111110111020011?011??11101011000?1?00010001101?000????0

Staurotypus

1??1000010100(0 1)110120120?0111110010010010(01)010011100000010000?01010102110010212202010000101121?0011100200001112001000012010201100101021?0000000?0010?12110011000101100011??????000100?01001010011010101100010100210(01)?0??00?01000?0111?11?00000011100011101010021??0100000001?1?00?1?1?001111101111001011101021?111?10111020011?001??1110101100001?00010001????????????

Kinosternon_suburum_hippocrepis 1??10000101000110120120?11111100100100001010011101001010000?01010101010010212102000000101121?00111002000010120000001120100001011010210000000101010?12110011000100100011??????000100?00101110011010101100010100201100??00?01000?0111?110000000111001?????010021??0100000001?1?11?1?1?0011111011110???11101021?111110111020011?011??11111011000?1?00010001????00?0?0??

Dermatemys_mawii

1??10000101(01)00110020120?010111000011000010000111010000100020(01)1011101010010212101000000101121?0011000210000?1200100000201000110000102(01)00000000?0010?12110011001??????011??????000010?010010100000?0101100010100110100??00?01000?0111?1100000000?1000111000?1011??0010000001?1?100100000011110110?001011101020111??10111020011?011??00101?11000?1?0001000?001?0000?0??

Baptemys_wyomingensis 1??1000?10110?110020120?010111000011000000000111010000100021010101010?0010212101000?00101121?001(01)0002?000101200100001201120???????020000000010?1?0?121000111?1????????1??????100010?000001100110?01011000101001?00?0??00?010???0111?1100000000?100011100011011??1?00000001?1?001100000011?1?111???????????2?111??1?111020?11?011??1???1?1????0??0??10001???????0?0?0

Emarginachelys_cretacea 1??1000?1011001100201????10110000001000?00000111010000?00?21010101?10????021?102010?001??121?0011?002100010110110?0????1?00?0?????020??000001??0?0?121100110?0001100???????????????????????0011??01011??0???001?00?0??00??10??????1?1100001000?10001110?2?0??1??0?00000001?1?00???010??11???111?????????????1??????11102?????0?1??01????1???????0??1000?101????0?0?0

Chrysemys_picta

1??1000010110(01)110020110?01011100001100101000011101000000002001010200110010212102000000101121?0011000210000?120010010020101111000010200000000100010?12110011000001100011??????0000101110000100000?0100?100101001000?0??00?01000?0101?1100000000?100011100011011??1010000001?1?00001000011111011121110111010201111110111020011?011??00011111000?1?00010001????000?????

Emys_orbicularis 1??10000101000110020111101010100001100001000011101000000000?01010000010000212101000000101120?0011000210000?120010000120100101001010200000000101011?121100110?0001100011??????0000?0???????100000?0100?100101001000?0??00?01000?0101?11000010010100011100010011??0100000001?1?00100000??111101112101011101020111111?111020011?011??00?11?1100001?00010001100?0000?0?0

Geoclemys_hamiltonii 1??10000101000110120111101111100000100001010011101000000000?01010211110000212102000000101121?00110002100010120010000020103101001010200000000100010?12110011110001100011??????000010?010000100110?0100?100101001100?0??00?0100100111?1100000000?100011100001011??1110000001?1?00101000011111011121010111010201111110111020011?011??0001111100011?00010001????0000?1??

Testudo 1??1000010110011012011110100??00001100001010011101000000002001010200010000201201000000101121?0011000200000?120010010100100101001010200000000101010?12110011010001100011??????0000000000010100000?0100?100001001000?0??00?1100100111?1100000000?100011100001001??0010000001?1?00001000011111011121010111010201111110111020011?011??0001111100001?00000001????100????0

Gopherus_polyphemus

1??10000101100110020120?0100??0000110000(01)000011101000010002001010210010100211201000000101121?0010000210100?1200100001001000?11000102200000000?1010?12110011010001100011??????0000100010000100000?0100?100001001000?0??00?1100100111?1100000000?100011100001001??1010000001?1?00001000011111011121010111010201111110111020011?011??0001111000001?00000001001?100????0

Chelonoidis_sp.

1??10000101101(12)1012011110100??00001100000000011101000000002001010210010000201201000000101121?001000021000111200100001001000?1001010220(01)00000101010?12110011010001110011??????0000000000000100000?0100?000001001100?0??00?0100100101?1100001000?1000111000101?1??0210000001?1?00000000001101011121010111000201111200111020011?001??0001111000001?00010001001?00?????0

Platysternon_megacephalum

1??10000101000(12)11000111001011100100000000001011101001000001?01010100010010202201000000101121?000100020100101201100000201000011110102000000020?0110?121100111000001001?1??????0000001(01)01001100000?0100?000001001100?0??00?0100100101?1100001000?1000111000101?1??0210000001?1?00000000001101011121010111000201111200111020011?001??0001111000001?00010001101?00?0?0?0

Apalone_spinifera 1??10100101101110120120?11111110000100100010011110110010001?11111200010110212202100000101121?0020101?10?10?120010110110100000011010201000000100010?120000111000011001?1??????000100011100111?00120100?000101?1?001011000?0101??1??1???????11(01)0?1000113013101?1??0?00001????1???????????11010120?0010111000201?11110111020011?001??0011101110011?00010011100?0010?0?0

Pelodiscus_sinensis 1??10100101101110120120?11111110000100100010011110110010000?11111200010110212202100000101121?0021101?10?10?120010110110100010011010201000000100010?12000011100001100(01)11??????000100011100111?00120100?000101?1?001011000?0101??1??1???????1110?10001130131?1?1????00001????????????????110?0120?00?011100020??1??10????????????????????????0????????????101?0010?0?0

Lissemys_punctata 1??10100101101110120120?11111010000100100010011110110010000?11111200000110212202100000101121?0021101?00?20?120010110110100000011010201000000100010?12000011100001100001??????000?00???0?0011?00120100?000101?1?00100??00?0101??1??1???????1110?1000113013101?1??0?00001????1???????????11010120?0010111000201?1??10111020011?001??0111101110011?0??1001?101?001????0

Carettochelys_insculpta 1??10100101100110120120?11011100100100101000011110000010001?01111010010110210202000?01101121?0021101?10?11(01)120010110120113000111010201000010100010?12100011100001100011??????000010111100110100120000?00010100000100??00?01000?1?11???????1000?100011100010021??0100001????1???????????11010110?0010111000201111110111020011?001??0111101000011?0001002211000000?0?0

Allaeochelys_libyca 1??1??0?1?110?1101201???11????00?0?10???1????1???????????????????????????0211202000001101121?0???10???0?11(01)1201??1101201130001?0010201?0001?10?010?12100011100001100011????????????????????????????????????????????????????????????????????????????????????????????????????????????????????????????????0????????????????????????????????????????????????????????????

Chelus_fimbriatus

1??0101010110110(01)?20110?01011101??21000011?0011111000000000?01010000001?00202300??000011?120?101000???0000?200?00001132101012001200201000000100010?120001110?0010100011??????1001?0???????000111(03)0100?100001001100?0??00?01000?0101?1100000000?100011100011011??3110000010211001000001?1011111131000010100201011110111120011?101110001101100001?00010001101?0000?0?0

Phrynops_geoffroanus

0110111010110110(01)?20111011011101??21000011?0011101000000000?01010000001?00202300??000011?120?101000???0010?200?00001132101012001210201000000101010?121001100?0011100011??????1001?0?10?100000000?0100?10010110110100??00?01000?0111?1100000000?100011100011011??1010000010010000000001?1000111131000010000201011110111120011?101110001101100001?0001000100??0000?0?0

Elseya_dentata 01101110101101101?00111111011101??21000011?0011101000000002011010000001?00202300??000011?120?101000???0000?200?10000120100012001210201000000100010?1(12)1001110?00111001?1??????001000010011000000??0100?100?01001?1110??00?010???1?11?1?00000000?1000111000?1001??101000001001?00?000001?10?0111131000010?0020101??10111120011?1?11100??101??001??00010001110????0?0??

Chelodina 01101110101111110?20111001011101??21000011?0011101000000000?01010000001?00202300??100011?120?101000???0010?200?00001122100012001200201000000100010?121001100?00111001?1??????100100000000000000130100?100?0100111110??00?01000?0111?1?00000000?100011100011011??0110000010110000000001?1000111131000010100201011110111120011?101110001101110001?00010001100?0000?0?0

Araripemys_barretoi 1??11110101101110?20111001011100001100101100011101000000000???11??000?1?01202100??000011?120?102100???0?00?200????00000101012001210201000000100010?121001100?00111001?1??????100100000000010011140100?100101001001010?00?01000?1?11?1101001110?000011101111011??0110000011???000000101?100?1111000000101002??011110111?20011?1??????????11?00?1?0??1000101100000?0?0

Podocnemis 1??11100101101110?00121111011110001100001110011101000011002002110000001?11202300??100011?120?101101???0000?200?10000020100010001210201000000100010?120000101?0001100011??????000000011000010000(01)30100?10010100110100??00?01000?1?11?1100000000?00001110001101011101000001001(0 1)001000001?1000111100000010000201011110111120011?101110001101110011?00010001110?0000?0??

Pelomedusa_subrufa 1??11100101101110?20120?01011100001100101100011101000010002002110?0???1?11202300??100011?120?101000???0010?200?10000100100012101210201000000101000?121001110?0011100011??????0000?0?110000100000?0100?10010100110100??00?01000?1?11?1100000000?000011100011010111010000010010000000001?1000111100000010000201011110111020011?101110001101100011?00010001000?0000?0?0

Ordosemys_sp._IVPP_V12092 0010000?1011011?0010111?0?0101000?1?001?0?00011101000000000?010102000?00102021011101(01)010111??00101002?00?101201?00100201?00??0011002?0?1000???1????1110?0011?000????1?1????????????????????????????????????????????????????????????????????????????????????????????010?????????????????????????????????0????????????????????????????????????????????????0???????????

Judithemys_sukhanovi ???1??0?101?01??0?101????101???0001?001000?00111??0?00?000??010102000?0??021210???0?001??121?001010001000101200100?0100100????????0????1000010101??1110?0111?000????1?1??????000100??????0?00000?0100?000101001?00?0??00?01001001?1?1100001000?1000112011?0001??0000000001?1?0000000(01)00?111011120(01)0001(01)0102?10112101110200?1?0?1??00??1?1000001?0??1000?????00?0???0

Annemys_levensis

001100001011011?00201110?101?10000200010(01)000011101000000000?010102000?0??0202101000000101110?0010?0001000??1101100001001000??01???020000010???????1?110?0?11?00011001?1011?0?000000?100000000000?0100?00010110110100??00?0100100111?1010101000?100011100010001??00000001000100000000100???????????????????0?00?????11102001010?1???0?????000001?0??????????????0?0??

Annemys_latiens 0010??1?101101??00201110?10???0?0???0???????01?1??0???0??????????????????????101000?0010011??0?1??0???0??????0??0???????0?1????????2???00?0???????0(01)110?0?11?0001100???????????????????????00000?0100?000101101?0(01)?0??00?0100100111?1(01)10101000?100011100010001??00000001000100000000100??????????????????????????????????????0???????????000001?0???????????????????

Annemys_sp._IVPP_V18106 0110001?10110110002011??01010100002000101000?11101000000000?010102000000102021010100?0101110?001000001000101101100?0?201000?10111?0210?00000101100?1110?0011100011001?1?????????????????????????????????????????????????????????????????????????????????????????????????????????????????????????????????????????????????????????????????????????????????????????????

Xinjiangchelys_wusu 0110?010101101?00020?????101??0000100010?00001110?0000??????01?1?2?00????0211101000?001??110?00101000100???1201100?0?201000???????0200?0000???????0(01)110?0111?00011001?1011???00???0?1????0000000?0?0??00010110110(01)?0??00?0100100111?1100101000?100011100010001??0000000000010000000010000???0????????1?0?????????????????????????????????0?0001?0??1000010100000?0??

Xinjiangchelys_radiplicatoides 0110??1?1011011?00?011????0???0?????0??????00111??00??0???????11?????????02111010?0?0?101111?0?2??0???0??1012?0?00001201000?001???0200000000201110?1110?0011000011001?1???????0?1?????????100000?0100?000???101?00?0??00?0100100111?1100???000?100011100010001??00000001000100000000100???????????????????0????????111?200101????????????000001????????????????0?0??

Dracochelys_bicuspis ???1000?101101??0??01????1010100001?0?????000111010001?0001?010100000?00?020?101010000101111?001010001000101201?0??0?2010?????????02???0000???????10110?0?11?0001100??1??????000?00?0?0?0010000??1100?000101001?00?10?00?0110101?0??1101?01100?1000????1??0001??0000000????????????????0111?110?000001000?0010????01110200???0???????????0?0001?0???000?????????????

Kirgizemys_hoburensis 0011000?10110110001011110101010?0020001010?0011101000000000?010102000?001020?101000000101111?0010?000100010110010010100100001001??02?0?100001010?0?1111?0110?0001100011??????0001?0????????00000?0100?000101101?00?0??00?010???0111?1100001000?100011201110001??0?00000001?1?0000000(01)10?111?111200000110??2?10?????????2???1?0?1?????????0?0001?????????????????????

Kirgizemys_dmitrievi ???1000?1011001?001?111??101010000200010000001110?0000?0001?000101000?00102021020?00001011?1?00100000?000????00100?0??01000??00???02?0?1000?1110???1111?0?1??00011101????????000100?000000????????????????????????????0??????????????????????????????20?1??????????????????????????????????????????????????????????????????????????????????????????????????????????0

Sinemys_gamera 01100?1?10110111002011??1101010??02100?000?0011???0??0?0??0?0????????????1202201000000101110?00?00000100010120110010?001000??00??00220?1000????????1110?0111?00011001?1????????????????????00001?0???????1?????100?10?0??010??????1??10???1????100???????1?00???00????0????1??????00?????????????????????????????????????????????????????001001?????????????????????

Sinemys_lens 0010??1?101101?100201????101??0??02?0000???00111??0000??0?????????0???????202201000?0?1??1?0?00100000?00010110110??????10?0???????022??1000????????1110?0111?0001100??1??????00????????????0000??0100?000101001100?10?00?0100101??1?1101001(0 1)00?1000112?1010001??00001000???1??0?0000?10?1?0?????0?????????0???1??????????????0?????????????????????1000?????000?????

Meiolania_planiceps 00000000101000101000110?000101000100110000000110010000000020010102100000102103110000(01)0101121?001(01)100000000?12001100000(01)11211100110020?0000000?101010111?0111100001101?11111110000000000100000000??100?00????001000?0??00??1????0101?0?00101100?0000110000?0011??000000000001000????????010001112000000000000?0022011110000101011??00001??000000?000000010?0?0000?0?0

Chubutemys_copelloi ???1000?101000?01?0011???10000000?000??00000011???0??000000?011102000????0202101?000001??110?0011000000001?010010000?00110???????0020??0000???????100???????????????1?1????????????????????00????0??????????0???0??????????0???????????0???????????????????????????????????????????????????1?1?????????0?????0?????1110?0???????????????????????????????????????????

Eubaena_cephalica 0000002?10100011002011???1011100001000000001011101000000001?0101020001000021210100000011?121?00101000?000101201100000201020??00???1210?0001010?110200????????00011011?1??????0000?0???????0?????????????????????????????????????????????????????????????????????????????????????????????????????????????????????????????????????????????????????????????????????????

Arundelemys_dardeni 0100001?1011001??020110?1?01?1??0???0????0?10?1101000000001?0101000000?0?02??101000000101111?001010001000111201?000000010200101110020?000000101110200???????0000????1?1?????????????????????????????????????????????????????????????????????????????????????????????????????????????????????????????????????????????????????????????????????????????????????????????

Pleurosternon_bullockii 0110001?1011001001001?1?110111000011000000010111010000?0000?01??00000????020110110000010111??0020?00010?0101101?00?0??????01100111022110001010111(01)200???????00001100??1????????????????????0000130??0?0?0101001?00?0??00?01000?1?11?0100100000?00001?1000??010101000000010010000000000000???0??????????0?000?????????????????0?1??00????????????????????????????????

Glyptops_plicatulus 01100?1?1011001?0?0011???101?100?0100000?0010111???0?0?00?1?0101000?0????0201101?00000101111?0020?00010?0101101??0?002?100?1100???0211?000101011?0200???????0000??????1??????00?0??0????0?00000100100?000101001?00?0??00?0100100111?0100100000?0000111000110101010000000000100000000000?00010??????????0?000101???011112001010?1??00??1?10000?1?0???????????????????

Kallokibotion_bajazidi 0000??0?10110010100011???10???00000000000??001100100000?00??110102??0????0202102000?00101111??0??00???0001?1200?00?00?1112111000101200000000101111?10???????000011001?1??????00?1???????00?00000?0000?000101???000?0??00?010011??1??00?000?000?0000111000??00011?20000000001000?0000000?00000????????0?0?????????001110100101011??0000???000000?????????????????????

Eileanchelys_waldmanni 00000?1?10110??0??001????1011?0000?00??0?00???11010000??0???01?100000????020?100?000001????0?????00???0?0??01010?00000(01)1000010?01002010?000?????????????????100011001?1??????10????????????00??0?0????000001101?00?0??00?01001001?1?0000????00?00001?0000?????????0???0?00???00?0????00????????????????????????????????????????????????????????????????????????????0

Kayentachelys_aprix 00000010101100101?00000?110111000000000000000?110?000000001?010100000????0201100??0010100010?0010000000000?0101000?0?001000??0???001?101000?11111(01)000????????0001100??0??????1000?0?000000000000?0000?00000000?100?0??00?00000?0111?0000001000?0000010000?000010000000000001000000000000?0?00??????????0000000100001010?00101011??00001?10?000??????000????????0?1??

Australochelys_africanus ????10??000??0101?00000?1?0????0000??000?0000?10??00?000001?0?01?01000???01?00?0??000?1??110?00?0000000000?00000?01??0?0??0????0?010?1?00?0?211?10000?????????????????0?????????????????????????????????????????????????????????????????????????????????????????????????????????????????????????????????????????????????????????????????????????????????????????????

Proganochelys_quenstedti 0000100?000000101?00000?0001010000000000?010001000000000001?000000100100000??0?0??00000??000?0010000000000?000?0001000000?0??0?0?000?0?0000?200100000????????0001010??0??????000000000000000000(01)00000??0?00000000100??00???0???010000?00?00000?0000000000?0??0100?0000000000100000000??010000????????000000000000000000000000010??00000?0000000?000000000?0?0000?1?0

Adocus_lineolatus 1??1000?101100110120120??10111000011001010000111010000100021010102000?0010202102010?00101121?00111002100010120110000?1011?0???????020000000010?1?0?111000111?00011000?1??????000?101110001100000?0100?101101001?0(01)?0??00?010???0111?1100100000?100011100010001??000000010001000101000101111?100?00101110102?111????111020011?011??0101111000001?0??10001001?????????

Petrochelys_kyrgyzensis ?????????0?????101201???????????????0????????1????????????????1??????????02?22021?00001???21???2?101??0??0?120010?101101000000??0?020100000??????0?1200?0111?00011001?1?????????????????????????????????????????????????????????????????????????????????????????????????????????????????????????????????????????????????????????????????????????????????????????????

Galianemys_whitei 1??11100101101110?2012101101110010010000110101110100001(01)000?011100000?1?10202200??101011?120?101001???0010?200?0001010010001000?11021100000210?010?120000011?00111001?1?????????????????????????????????????????????????????????????????????????????????????????????????????????????????????????????????????????????????????????????????????????????????????????????

Leviathanochelys_aenigmatica ???????????????????????????????????????????????????????????????????????????????????????????????????????????????????????????????????????????????????????????????????????????????????????????1-000-0???????101?????0?1??0??????????????????????????????????????????????????????????????????????????????????????????????????????011110?1???????????????????????????????

;

END;

BEGIN ASSUMPTIONS;

TYPESET * UNTITLED = unord: 1- 356;

END;

BEGIN MESQUITECHARMODELS;

ProbModelSet * UNTITLED = 'Mk1 (est.)': 1- 356;

END;

Begin MESQUITE;

MESQUITESCRIPTVERSION 2;

TITLE AUTO;

tell ProjectCoordinator;

timeSaved 1659257748607;

getEmployee #mesquite.minimal.ManageTaxa.ManageTaxa;

tell It;

setID 0 2172096337510380182;

tell It;

setDefaultOrder 0 1 2 3 4 5 6 7 8 9 10 11 12 13 14 15 16 17 18 19 20 21 22 24 25 26 27 28 29 30 31 32 33 34 35 36 37 38 39 40 41 42 43 44 45 46 47 48 49 50 51 52 53 54 55 56 57 58 59 60 61 62 63 64 65 66 67 68 69 70 71 72 73 74 75 76 77 78 79 80 81 82 83 84 85 86 87 88 89 90 91 92 93 94 95 96 23;

attachments ;

endTell;

endTell;

getEmployee #mesquite.charMatrices.ManageCharacters.ManageCharacters;

tell It;

setID 0 5384630264835292583;

mqVersion 370;

checksumv 0 3 970652197 null getNumChars 356 numChars 356 getNumTaxa 97 numTaxa 97 short true bits 31 states 31 sumSquaresStatesOnly 65218.0 sumSquares 65218.0 longCompressibleToShort false usingShortMatrix true NumFiles 1 NumMatrices 1;

mqVersion;

endTell;

getWindow;

tell It;

suppress;

setResourcesState false false 100;

setPopoutState 300;

setExplanationSize 0;

setAnnotationSize 0;

setFontIncAnnot 0;

setFontIncExp 0;

setSize 1707 841;

setLocation -8 0;

setFont SanSerif;

setFontSize 10;

getToolPalette;

tell It;

endTell;

desuppress;

endTell;

getEmployee #mesquite.charMatrices.BasicDataWindowCoord.BasicDataWindowCoord;

tell It;

showDataWindow #5384630264835292583 #mesquite.charMatrices.BasicDataWindowMaker.BasicDataWindowMaker;

tell It;

getWindow;

tell It;

getTable;

tell It;

rowNamesWidth 133;

endTell;

setExplanationSize 30;

setAnnotationSize 20;

setFontIncAnnot 0;

setFontIncExp 0;

setSize 1607 769;

setLocation -8 0;

setFont SanSerif;

setFontSize 10;

getToolPalette;

tell It;

setTool mesquite.charMatrices.BasicDataWindowMaker.BasicDataWindow.ibeam;

endTell;

setActive;

setTool mesquite.charMatrices.BasicDataWindowMaker.BasicDataWindow.ibeam;

colorCells #mesquite.charMatrices.NoColor.NoColor;

colorRowNames #mesquite.charMatrices.TaxonGroupColor.TaxonGroupColor;

colorColumnNames #mesquite.charMatrices.CharGroupColor.CharGroupColor;

colorText #mesquite.charMatrices.NoColor.NoColor;

setBackground White;

toggleShowNames on;

toggleShowTaxonNames on;

toggleTight off;

toggleThinRows off;

toggleShowChanges on;

toggleSeparateLines off;

toggleShowStates on;

toggleReduceCellBorders off;

toggleAutoWCharNames on;

toggleAutoTaxonNames off;

toggleShowDefaultCharNames off;

toggleConstrainCW on;

toggleBirdsEye off;

toggleColorOnlyTaxonNames off;

toggleShowPaleGrid off;

toggleShowPaleCellColors off;

toggleShowPaleExcluded off;

togglePaleInapplicable on;

togglePaleMissing off;

toggleShowBoldCellText off;

toggleAllowAutosize on;

toggleColorsPanel off;

toggleDiagonal on;

setDiagonalHeight 80;

toggleLinkedScrolling on;

toggleScrollLinkedTables off;

endTell;

showWindow;

getWindow;

tell It;

forceAutosize;

endTell;

getEmployee #mesquite.charMatrices.AlterData.AlterData;

tell It;

toggleBySubmenus off;

endTell;

getEmployee #mesquite.charMatrices.ColorByState.ColorByState;

tell It;

setStateLimit 9;

toggleUniformMaximum on;

endTell;

getEmployee #mesquite.charMatrices.ColorCells.ColorCells;

tell It;

setColor Red;

removeColor off;

endTell;

getEmployee #mesquite.categ.StateNamesStrip.StateNamesStrip;

tell It;

showStrip off;

endTell;

getEmployee #mesquite.charMatrices.AnnotPanel.AnnotPanel;

tell It;

togglePanel off;

endTell;

getEmployee #mesquite.charMatrices.CharReferenceStrip.CharReferenceStrip;

tell It;

showStrip off;

endTell;

getEmployee #mesquite.charMatrices.QuickKeySelector.QuickKeySelector;

tell It;

autotabOff;

endTell;

getEmployee #mesquite.charMatrices.SelSummaryStrip.SelSummaryStrip;

tell It;

showStrip off;

endTell;

getEmployee #mesquite.categ.SmallStateNamesEditor.SmallStateNamesEditor;

tell It;

panelOpen true;

endTell;

endTell;

endTell;

endTell;

end;
